# Upregulated NMDAR-mediated GABAergic transmission underlies autistic-like deficits in *Htr3a* knockout mice

**DOI:** 10.7150/thno.60531

**Published:** 2021-09-07

**Authors:** Lang Huang, Jing Wang, Guanmei Liang, Yue Gao, Shi-Yang Jin, Jian Hu, Xiaoxue Yang, Jianpei Lao, Jinfa Chen, Zhou-Cai Luo, Cuixia Fan, Li Xiong, Xinhong Zhu, Tian-Ming Gao, Mei Zhong, Xinping Yang

**Affiliations:** 1 Center for Genetics and Developmental Systems Biology, Department of Obstetrics & Gynecology, Nanfang Hospital, Southern Medical University, Guangzhou 510515, China.; 2 Key Laboratory of Mental Health of the Ministry of Education, Guangdong-Hong Kong-Macao Greater Bay Area Center for Brain Science and Brain-Inspired Intelligence, Guangdong Key Laboratory of Psychiatric Disorders, Collaborative Innovation Center for Brain Science, Department of Neurobiology, School of Basic Medical Sciences, Southern Medical University, Guangzhou 510515, China.; 3 Department of Bioinformatics, School of Basic Medical Sciences, Southern Medical University, Guangzhou 510515, China.; 4 Institute of Neuroscience and Department of Neurology, The Second Affiliated Hospital of Guangzhou Medical University, Guangzhou 510260, China.

**Keywords:** *Htr3a*, autism, transcriptome, NMDAR, interactome

## Abstract

Mutations in serotonin pathway genes, especially the serotonergic receptor subunit gene *HTR3A*, are associated with autism. However, the association of *HTR3A* deficiency with autism and the underlying mechanisms remain unknown.

**Methods:** The *Htr3a* knockout (KO) mice were generated using transcription activator-like effector nuclease technology. Various behavior tests, including social interaction, social approach task, olfactory habituation/dishabituation, self-grooming, novel object recognition, contextual fear conditioning, elevated plus maze, open field and seizure susceptibility, were performed to assess the phenotypes. Transcriptome sequencing was carried out to search for molecular network and pathways underlying the phenotypes. Electrophysiological recordings, immunoblotting, immunofluorescence staining, immunoprecipitation, and quantitative real-time PCR were performed to verify the potential mechanisms. The N-methyl-D-aspartate receptor (NMDAR) antagonist memantine was used to treat the KO mice for rescuing the phenotypes.

**Results:** The* Htr3a* KO mouse model showed three phenotypic domains: autistic-like behaviors (including impaired social behavior, cognitive deficits, and increased repetitive self-grooming), impaired memory, and attenuated susceptibility to pentylenetetrazol-induced seizures. We observed enhanced action potential-driven γ-aminobutyric acid-ergic (GABAergic) transmission in pyramidal neurons and decreased excitatory/inhibitory (E/I) ratio using the patch-clamp recording. Transcriptome sequencing on the hippocampus revealed the converged pathways of the dysregulated molecular networks underlying three phenotypic domains with upregulation of NMDAR. We speculated that *Htr3a* KO promotes an increase in GABA release through NMDAR upregulation. The electrophysiological recordings on hippocampal parvalbumin-positive (PV^+^) interneuron revealed increased NMDAR current and NMDAR-dependent excitability. The NMDAR antagonist memantine could rescue GABAergic transmission in the hippocampus and ameliorate autistic-like behaviors of the KO mice.

**Conclusion:** Our data indicated that upregulation of the NMDAR in PV^+^ interneurons may play a critical role in regulating GABAergic input to pyramidal neurons and maybe involve in the pathogenesis of autism associated with *HTR3A* deficiency. Therefore, we suggest that the NMDAR system could be considered potential therapeutic target for autism.

## Introduction

Autism spectrum disorder (ASD), prevalent in 0.75% to 1.1% of the population, is a class of neurodevelopmental disorders with core features of impaired social interaction and communication difficulties, along with stereotyped behaviors and restricted interests 1, 2. Apart from these core features, most of individuals with ASD show a lower level of intelligence quotient (IQ) 3 and comorbidity with epilepsy (EP) 2.

The serotonergic, γ-aminobutyric acid-ergic (GABAergic) and glutamatergic systems are reported to be involved in ASD 4, 5. Multiple lines of evidence indicate that ASD is associated with mutations in many genes that affect the ratio between neuronal excitation and inhibition 6-9. For instance, mutations underlying tuberous sclerosis 10, fragile X syndrome 11 and Angelman syndrome 9, targeting proteins critical for synaptic functions, have been associated with unbalanced excitation and inhibition. Both upregulation 12 and downregulation 13 in the ratio of excitation to inhibition were observed in the brains of autistic mouse models. The serotonergic system is shown to play a critical role in maintaining the balance of excitatory/inhibitory (E/I) transmission to keep proper functions of neuronal networks in the brain 14-17.

Human brain development undergoes high serotonin synthesis during childhood, and dysregulation of the developmental process is reported in autistic children 18, 19. Elevation of serotonin levels in the whole blood and platelets is detected in nearly 30% of individuals with ASD 20, 21. The serotonin transporter gene (*Sert*) knockout mice show hyperserotonemia and autistic-like behaviors 21. The serotonin transporter gene *SLC6A4* has also been implicated in ASD 22, 23. The *TPH2* gene, which encodes the rate-limiting enzymes that control serotonin biosynthesis and is associated with autism 24-26. Mutations within genes in the serotonin pathways, especially the serotonergic receptor subunit gene *HTR3A,* have been reported to associate with autism 27, 28.

The 5-HT3 receptor consists of the five subunits 5-HT3A-E, of which the subunits 5-HT3A-C are expressed in the brain 29. The 5-HT3A subunit is essential for all the functional 5-HT3 receptors, mainly expressed in the interneurons of the cerebral limbic system, including the hippocampus, cortex, and amygdala 30, 31. Previous studies have reported impaired social behaviors 32, decreased anxiety 33, and impaired fear memory extinction 34 in *Htr3a* knockout mice. These studies on *Htr3a* knockout mouse models focused on different behavior aspects. Since previous studies indicated that *HTR3A* might be involved in human autism, a systematic evaluation of autistic-like behaviors of mice with the knockout of this gene is needed, and elucidation of the underlying molecular pathways would shed light on the pathogenesis of autism involving* HTR3A* mutations.

Here, we generated *Htr3a* knockout (KO) mice, displaying autistic-like behaviors, impaired learning/memory (LM) and attenuated susceptibility to seizures. Electrophysiological recordings revealed a decreased excitatory/inhibitory (E/I) ratio caused by the enhancement of action-potential-driven GABAergic input to pyramidal neurons. We carried out transcriptome sequencing on the hippocampus and systematically searched for molecular pathways by integrating the transcriptomics and protein interaction data. The converged pathways associated with different behavioral phenotypes indicated upregulation of N-methyl-D-aspartate receptor (NMDAR) system in the mutants. Immunostaining of GluN2B and parvalbumin revealed an upregulation of NMDAR in the parvalbumin (PV^+^) positive cells. The electrophysiological recording showed enhanced NMDAR current and excitability in PV^+^ interneurons, and the NMDAR antagonist D-APV reduced their excitability. Intraperitoneal (i.p.) injection with memantine (an NMDAR inhibitor) reversed the abnormal behaviors and spontaneous inhibitory postsynaptic currents (sIPSCs) of the *Htr3a* KO mice. These results suggested that NMDAR upregulation in PV^+^ interneurons increased excitability and GABAergic output, possibly contributing to the imbalance of excitation and inhibition and leading to autistic-like behaviors.

## Materials and Methods

### Animals

The generation of *Htr3a* KO C57BL/6N mice by transcription activator-like effector nuclease (TALEN) technology were generated by Cyagen Biosciences (China). Briefly, 11 bases (GGGGAAGgtaa) in exon 1 of the mouse *Htr3a* gene (Ensemble ID: ENSMUSG00000032269; Gene ID: 15561) were chosen as TALEN target sites. The 11-base deletion across the junction of the first exon and intron disrupted the reading frame after the 16^th^ codon in all the isoforms based on the UCSC genome browser (https://genome.ucsc.edu/). TALEN mRNAs transcribed *in vitro* and injected into fertilized eggs for KO mouse production. The genomic region spanning the deletion was PCR-amplified using *forward primer* 5'-AGTTGGAAAAGCAGTCTGCCTGG-3' and *reverse primer* 5'-TTGACCCCACACCTCAGAATCCT-3', followed by Sanger sequencing for genotyping. Age-matched (8-12 weeks) male or female mice and the wild-type (WT) littermates of the KO mice were used for behavioral tests, and male mice were used for all other tests.

### Behavior tests

Procedures for behavior tests are described in Supplementary Information, including the animal housing and handling, home cage social interaction test, social approach task, olfactory habituation/dishabituation test, self-grooming test, novel object recognition, contextual fear conditioning, elevated plus maze test, open field test, seizure susceptibility test and memantine rescue experiments. All experimental procedures on animals followed the guidelines and recommendations of the Institutional Animal Care and Use Committee (IACUC) and were approved by the Southern Medical University Experimental Animal Ethics Committee.

### RNA-seq and differential expression analysis

Each RNA sample was extracted from the hippocampi of adult mice according to the manufacturer's protocol (RNAeasy Mini Kit, Qiagen, USA). The quality and yield of the isolated RNAs were assessed using a NanoDrop Spectrophotometer (Thermo Fisher Scientific, Waltham, MA, USA) and Agilent 2100 Bioanalyzer (Agilent Technologies, Santa Clara, CA, USA). Differentially expressed genes (DEGs) were the intersection part of DESeq2 (*adj.p* < 0.05) and edgeR (*p <* 0.01).

### Immunoblotting and immunoprecipitation

The hippocampal tissue from 8-week mice was lysed in lysis buffer with 1 mM PMSF and centrifugated at 14,000 g for 15 min. For immunoprecipitation, the supernatant lysate was incubated with rabbit anti-GluN1 (1:50, 5704S, Cell Signaling Technology, USA) or IgG antibody (1:500, 2729S, Cell Signaling Technology, USA) at 4 °C overnight. Then the mixture was incubated with Protein A/G plus-Agarose (SC-2003, Santa Cruz, USA) for 3 h at 4 °C. After centrifugation at 1000 g for 5 min at 4 °C, the immunoprecipitates were washed three times with lysis buffer, and then boiled in protein loading buffer for 5 min. Western blotting was performed to detect the proteins using the following primary antibodies at 1:1000 dilution: rabbit anti-GluN1 (5704S, Cell Signaling Technology, USA), rabbit anti-GluN2A (ab124913, Abcam, UK), rabbit anti-GluN2B (14544S, Cell Signaling Technology, USA), rabbit anti-PSEN1 (6543S, Cell Signaling Technology, USA), anti-AMPAR1 (ab109450, Abcam, UK), rabbit anti-AMPAR2 (ab206293, Abcam, UK).

### Immunostaining

Mice were anesthetized with isoflurane and then perfused with ice-cold saline, followed by 4% paraformaldehyde (PFA) in 0.1 M phosphate-buffered saline (PBS), pH 7.4. After post-fixation overnight in 4% PFA at 4 °C, brains were transferred to 30% sucrose in 0.1 M PBS, pH 7.4. Coronal sections (40 µm) of the hippocampus were obtained on a cryostat (Leica CM3050 S) and rinsed in 1% TritonX-100 in PBS. The hippocampus sections were incubated in blocking buffer (containing 1% TritonX-100, 1% bovine serum albumin and 10% goat serum in PBS) for 2 h and then with following primary antibodies in blocking buffer for 48 h at 4 °C: rabbit anti-GluN2B (ab65783, Abcam, UK; 1:200) and mouse anti-PV (PV 235, Swant, Switzerland; 1:3,000). After washing three times, the sections were incubated with appropriate secondary antibodies Alexa Flour 594-conjugated anti-mouse IgG (1:1,000; Thermo Fisher, USA) or Alexa Fluor 488-conjugated anti-rabbit IgG (1:1,000; Thermo Fisher, USA) for 2 h. After three more washes in PBS, sections were mounted with the Pro-Long anti-fade medium (Invitrogen). Fluorescent images were collected using a confocal microscope (Nikon A1) and analyzed using the Image J software.

### qRT-PCR

Total mRNAs from hippocampal tissues were extracted using standard column purification according to the manufacturer's protocol (RNAeasy Mini Kit, Qiagen, USA), and reverse-transcribed into cDNAs using the PrimeScriptTM RT reagent Kit following the manufacturer's protocol (Takara, code No. RR037A). The qRT-PCRs were carried out using Applied Biosystems ABI 7500. The housekeeping gene *Gapdh* was used as the internal control. The relative expression levels of the genes were calculated using the 2^-∆∆CT^ method. The primers used for mRNA quantification are listed in Supplementary Information.

### Slice preparation

Mice were anesthetized with isoflurane and then decapitated. The brain was quickly isolated and transferred to ice-cold oxygenated artificial cerebrospinal fluid (ACSF) containing 220 mM sucrose, 2.5 mM KCl, 1.3 mM CaCl_2_, 2.5 mM MgSO_4_, 1 mM NaH_2_PO_4_, 26 mM NaHCO_3_, and 10 mM glucose. The coronal hippocampal slices (300 μm) were prepared using VT-1200S vibratome (Leica, Germany). The hippocampal slices were incubated in ACSF containing 126 mM NaCl, 3 mM KCl, 1.25 mM NaH_2_PO_4_, 1.0 mM MgSO_4_, 2.0 mM CaCl_2_, 26 mM NaHCO_3_, and 10 mM Glucose at 34 °C for 30 min. Then slices were put at room temperature (25 ± 1 °C) for 2 to 8 h. All extracellular solutions were constantly carbonated (95% O_2_, 5% CO_2_).

### Electrophysiology

Whole-cell patch-clamp recordings of the postsynaptic currents and excitability on hippocampal neurons were carried out as previously described 35. During the procedure, the recording chamber was continuously perfused with ACSF (2 mL/min) saturated with 95% O_2_/5% CO_2_ at 32-34 °C. The postsynaptic currents and excitability of the hippocampal neurons were recorded using MultiClamp 700B amplifier and 1440A digitizer (Molecular Devices) under IR-DIC visualization (Zeiss, Axioskop 2). The glass pipettes (resistance of 3-6 MΩ) were pulled with a micropipette puller (P-97, Sutter Instrument). Spontaneous excitatory postsynaptic currents (sEPSCs) were recorded at the reversal potential of GABA_A_ receptors (-70 mV) in the presence of bicuculline (20 μM). Glass pipettes were filled with the intracellular solution containing 130 mM potassium gluconate, 20 mM KCl, 10 mM HEPES, 4 mM Mg-ATP, 0.3 mM Na-GTP, 10 mM disodium phosphocreatine, and 0.2 mM EGTA, pH 7.2, 295 mOsm. For the neuronal excitability recording, the pipette solution was the same as sEPSCs recording. Neurons were held at -70 mV, and the spike discharges were induced by injection of hyperpolarizing and depolarizing current steps. Spontaneous inhibitory postsynaptic currents (sIPSCs) were recorded at the reversal potential of ionotropic glutamate receptors at a holding potential of 0 mV. Glass pipettes were filled with the intracellular solution containing 110 mM Cs_2_SO_4_, 0.5 mM CaCl_2_, 2 mM MgCl_2_, 5 mM EGTA, 5 mM HEPES, 5 mM tetraethylammonium, and 5 mM ATP-Mg, pH 7.2, 292 mOsm. For miniature postsynaptic current recording, tetrodotoxin (1 μM) was included in the perfusion solution. For evoking EPSCs, the stimulating electrode was placed in the DG fiber path, approximately 0.2 mm away from the recorded cell bodies in the CA1. The internal solution contained 115 mM CsMeSO_4_, 20 mM CsCl, 10 mM HEPES, 2.5 mM MgCl_2_, 10 mM sodium phosphocreatine, 5 mM QX-314, 4 mM Na-ATP, 0.4 mM Na-GTP, and 0.6 mM EGTA, pH 7.3, 285 mOsm. AMPAR-mediated currents were recorded at -70 mV, and NMDAR-mediated currents were voltage-clamped at +40 mV and quantified by measuring the amplitude current at 50 ms after stimulation. The E/I ratio was calculated by recording sEPSCs and sIPSCs on the same cell at -70 mV and 0 mV, respectively, with the internal solution containing: 130 mM CsMeSO_4_, 10 mM NaCl, 10 mM EGTA, 4 mM Mg-ATP, 0.3 mM Na-GTP, 10 mM HEPES, 290 mOsm, adjusted to pH 7.4 with CsOH. The E/I ratio was calculated as the frequency of sEPSC divided by the frequency of sIPSC per cell. Currents were filtered at 3 kHz with a low-pass filter, and data were digitized at 10 kHz and acquired using the pCLAMP 10 software. Series resistance was continuously monitored for each neuron. The neuron recordings were discarded if the variation of series resistance was over 20%. Data analyses were conducted by the Mini Analysis software (Synaptosoft).

### Statistical analysis

Statistical comparisons were conducted in GraphPad Prism 8. The homoscedasticity and normality of the distributions of data were determined using GraphPad Prism 8 before assigning specific statistical tests. Where normality and equal variance between samples groups were achieved, Student's *t* test, one-way analysis of variance (ANOVA) or two-way (ANOVA) were used. Where normality or equal variance of samples failed, Mann Whitney test or Kruskal-Wallis one-way ANOVA was performed. Most data were presented using box and scatterplots depicting the median, 5%-95% confidence intervals and individual values. Some data were expressed as the means ± standard errors of the means (SEM). Significance was conventionally set as **** *p* < 0.0001, ***p < 0.001, **p < 0.01 and *p < 0.05.

## Results

### Autistic-like behaviors, impaired memory and attenuated susceptibility to seizures in* Htr3a* KO mice

We generated an 11-base (GGGGAAGgtaa) frame-shift deletion in the first exon of *Htr3a* in C57BL/6N mice using the TALEN technology (**Figure S1A**), disrupting the reading frame after the 16^th^ codon in all the isoforms based on the UCSC genome browser (https://genome.ucsc.edu/). The ablation of *Htr3a* transcripts in the brains of knock out mice was determined by performing qRT-PCR on total RNA from the hippocampus. The forward primer was located at the deletion site in the first exon (3' end in the deletion region) and the reverse prime was in the common region of the isoform transcripts. The *Htr3a* transcripts were present in the hippocampus from WT, but were not detected in the tissue from *Htr3a^-/-^* mice (Student's *t* test, *p =* 0.0014) (**Figure S1B**).

**Figure 1:**
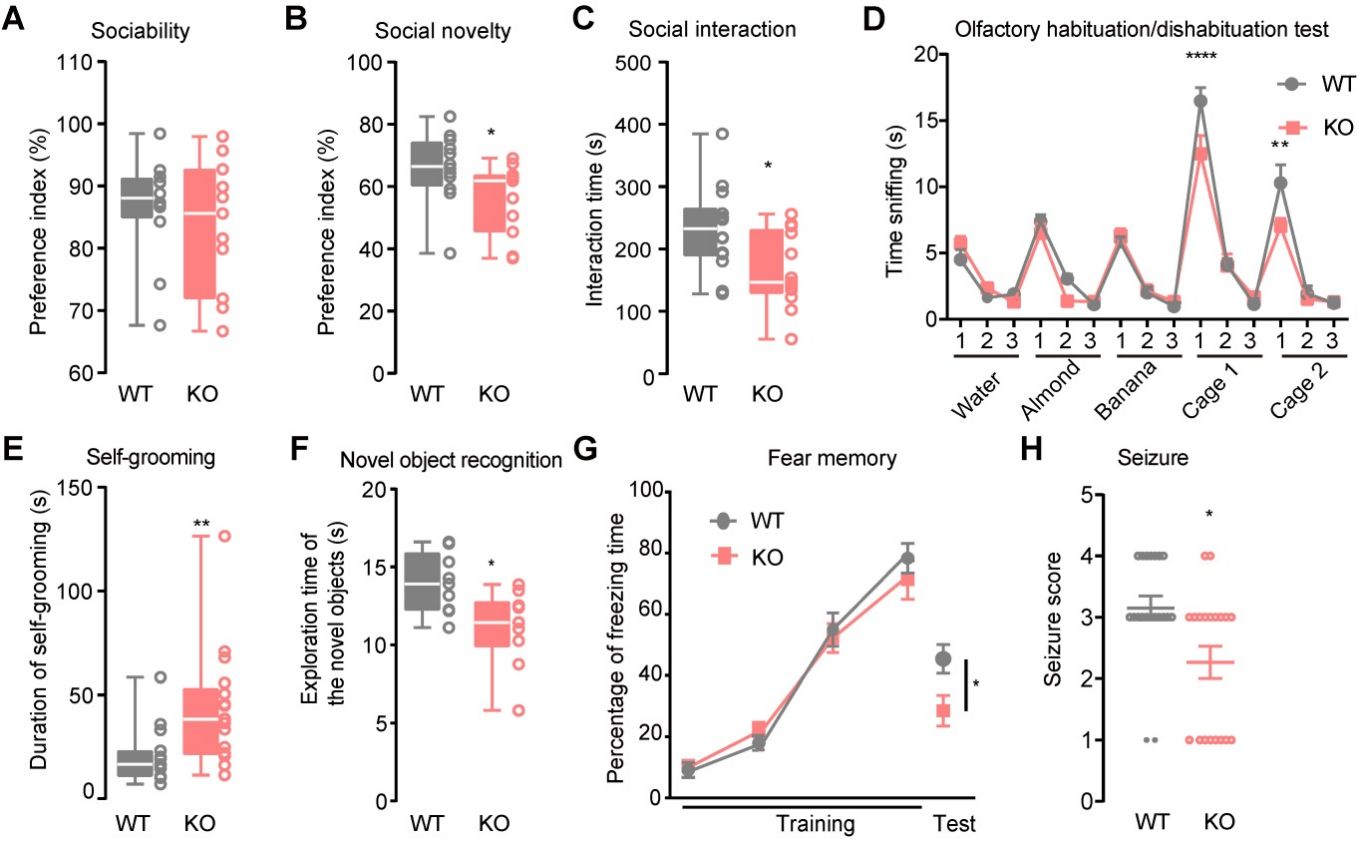
** Autistic-like behaviors, impaired memory, and attenuated susceptibility to pentylenetetrazol (PTZ)-induced seizures in *Htr3a* KO mice. (A)** In the 10-min sociability phase of the social approach task, sniffing time was used to calculate social preference index: T_S1_/(T_S1_+T_O_), T_S1_ - time for a testing mouse interacting with a stranger mouse (S1, Stranger 1), T_o_ - time for a testing mouse interacting with an empty cage (O, Object). There was no significant difference in social preference between the KO mice and WT mice (Mann Whitney test, *p* = 0.5254, *n* = 12 for WT mice, *n* = 11 for *Htr3a^-/-^* mice). **(B)** In the 10-min social novelty phase of the social approach task, sniffing time was used to calculate social preference index: T_S2_/(T_S1_+T_ S2_), T_S1_ - time for a testing mouse interacting with a familiar mouse (S1, Stranger 1), T_S2_ - time for a testing mouse interacting with stranger 2 mouse (S2, Stranger 2). Compared with WT mice, the *Htr3a^-/-^* mice showed significantly decreased preference to interact with stranger 2 mouse (S2, Stranger 2) over a familiar mouse (S1, Stranger 1), (Student's *t* test, *p* = 0.0448, *n* = 12 for wild type mice, *n* = 11 for *Htr3a^-/-^* mice). **(C)** The *Htr3a^-/-^* mice showed less active interaction time than WT mice in the 10-min social interaction test (Student's *t* test, *p* = 0.0125, *n* = 14 for WT mice, *n* = 13 for *Htr3a^-/-^* mice). **(D)** Different odors were presented to mice for three times in the olfactory habituation/dishabituation test. The *Htr3a^-/-^* mice spent less time sniffing social odors compared to WT mice. Statistic tests: (1) two-way ANOVA, odor effect F_14,240_ = 77.70, *p* < 0.0001; genotype effect F_1,240_ = 4.646, *p* = 0.0321; interaction between odor and genotype F_14,240_ = 2.968, *p* = 0.0003; (2) Bonferroni's multiple comparisons test, *p* < 0.0001 for the first test of the mouse cage 1; *p* = 0.0023 for the first test of mouse cage 2, *n* = 9 mice for each genotype. **(E)** *Htr3a^-/-^* mice spent more time on self-grooming than WT mice (Mann Whitney test, *p =* 0.0047, *n* = 15 for WT mice, *n* = 17 for *Htr3a^-/-^* mice). **(F)** In the novel object recognition test, the *Htr3a^-/-^* mice spent less time exploring the novel object than WT mice (Student's *t* test, *p =* 0.0122, *n* = 9 for WT mice, *n* = 10 for *Htr3a^-/-^* mice). **(G)** In the contextual fear conditioning test, both genotypes showed a similar percentage of freezing time in the 4 times of the tone-shock paired training (two-way ANOVA test, F_3,56_ = 0.7417, *p* = 0.5317). After 24 h, the KO mice showed less freezing time in the contextual fear memory test than WT mice (Student's *t* test, *p* = 0.0269, *n* = 8 for WT mice, *n* = 8 for *Htr3a^-/-^* mice). **(H)** The *Htr3a^-/-^* mice showed reduced seizure scores after PTZ injection (Mann Whitney test, *p* = 0.0115, *n* = 20 for WT mice, *n* = 19 for *Htr3a^-/-^* mice). Data are presented as boxplots (median and 5^th^-95^th^ percentile whiskers), or mean ± SEM., * *p* < 0.05; ** *p* < 0.01, *** *p* < 0.001, **** *p* < 0.0001.

In the three-chamber social approach task test, both *Htr3a^-/-^* and WT mice had a significant preference for sniffing or exploring a cage containing a stranger mouse (stranger 1) rather than an empty cage (For male mice, Mann Whitney test, *p* = 0.5254,** Figure 1A, Figure S1C**; for female mice, Student's *t* test, *p =* 0.2426, **Figure S2A**). However, compared with the WT controls, *Htr3a^-/-^* mice showed less preference for sniffing or exploring the stranger 2 mouse rather than the familiar stranger 1 mouse (For male mice, Student's *t* test, *p =* 0.0448,** Figure 1B, Figure S1C**; for female mice, Student's *t* test, *p =* 0.0198, **Figure S2B**). In the home cage social interaction test, *Htr3a^-/-^* mice spent shorter time engaged in exploring a stranger mouse, an active social interaction, than WT littermates (For male mice, Student's *t* test, *p =* 0.0125,** Figure 1C**; for female mice, Student's *t* test, *p =* 0.0116, **Figure S2C**). In the olfactory habituation/dishabituation test, both WT and *Htr3a^-/-^* mice demonstrated normal ability to habituate or dishabituate to odors. However, the *Htr3a^-/-^* mice spent less time sniffing the social odors than WT mice (For male mice, two-way ANOVA, Bonferroni's multiple comparisons test, for cage 1 (1st), *p <* 0.0001, for cage 2 (1st) *p =* 0.0023, **Figure 1D**; for female mice, two-way ANOVA, Bonferroni's multiple comparisons test, for cage 1 (1st), *p <* 0.0001, for cage 2 (1st) *p <* 0.0001, **Figure S2D**). These observations indicated that the *Htr3a^-/-^* mice may have less interest in social odors. The male *Htr3a^-/-^* mice displayed longer total duration time in repetitive self-grooming (Mann Whitney test, *p =* 0.0047, **Figure 1E**), while the female* Htr3a^-/-^* mice displayed normal self-grooming duration (Mann Whitney test, *p =* 0.1128, **Figure S2E**). In the elevated plus-maze test, we found no difference in the time spent in the open arms between the WT and *Htr3a^-/-^* mice (For male mice, Student's *t* test, *p =* 0.8526, **Figure S1D;** for female mice, Student's *t* test, *p =* 0.8742, **Figure S2F**). In the open field test, the *Htr3a^-/-^* mice traveled a similar total distance compared with the WT controls (For male mice, Student's *t* test, *p* = 0.6452, **Figure S1E**; for female mice, Student's *t* test, *p =* 0.4041, **Figure S2G**), suggesting that the *Htr3a^-/-^* mice had normal locomotion.

The novel object recognition test was performed to assess the cognitive function. During the familiarization session, both *Htr3a^-/-^* and WT mice showed no preference for the object (For male mice, Student's *t* test, *p* = 0.9353, **Figure S1F**; for female mice, Student's *t* test, *p =* 0.4579, **Figure S2H**). During the test session, the *Htr3a^-/-^* mice spent less time sniffing a novel object than the familiar one (For male mice, Student's *t* test, *p =* 0.0122, **Figure 1F**; for female mice, Student's *t* test, *p =* 0.0060, **Figure S2H**), suggesting that the *Htr3a^-/-^* mice had impaired recognition memory. In the contextual fear conditioning test, the mutant mice showed a significantly reduced fear memory compared to the WT controls (For male mice, Student's *t* test, *p =* 0.0269, **Figure 1G**; for female mice, Student's *t* test, *p =* 0.0035, **Figure S2I**).

Since EP shows comorbidity with ASD 2, we assessed the susceptibility to seizures of the mutant mice by injecting of pentylenetetrazol (PTZ) at a dose of 60 mg/kg body weight. Of the 20 WT mice, 11 (55%) displayed clonus, 7 (35%) displayed clonic seizure. Of the 19 the *Htr3a^-/-^* mice, 9 (47.37%) displayed clonus and 2 (10.53%) displayed clonic seizure, demonstrating that the *Htr3a^-/-^* mice could have significantly less severity of PTZ-induced seizure than WT mice (Mann Whitney test, *p* = 0.0115) (**Figure 1H**). However, no difference was observed between the mutant and WT female mice in the PTZ-induced seizure test (Mann Whitney test, *p* = 0.2445) (**Figure S2J**).

### Enhanced action potential-dependent GABAergic transmission

**Figure 2:**
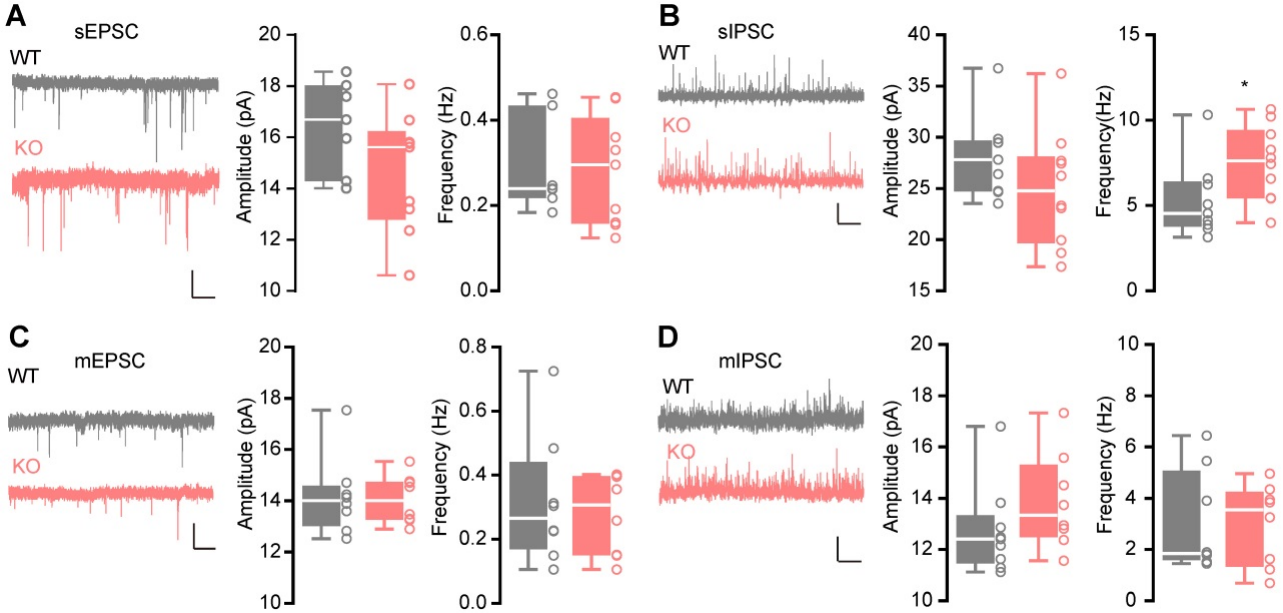
** Enhanced action potential-dependent GABAergic input of pyramidal neurons in the CA1 region of KO mice. (A)** Representative traces of recorded sEPSCs and boxplots of sEPSCs, showing similar sEPSC amplitude (Student's *t* test, *p* = 0.1108, *n* = 7 cells from 3 WT mice, *n* = 9 cells from 3 *Htr3a^-/-^* mice) and frequency (Mann Whitney test, *p* = 0.7577) in* Htr3a^-/-^* and WT mice. Scale bar: 20 pA, 2 s. **(B)** Representative traces of recorded sIPSCs and the boxplots of sIPSCs, showing normal sIPSC amplitude (Student's *t* test, *p* = 0.1866, *n* = 9 cells from 3 WT mice, *n* = 10 cells from 4 *Htr3a^-/-^* mice) and higher sIPSC frequency (Student's *t* test, *p* = 0.0422) in the* Htr3a^-/-^* and WT mice. Scale bar: 20 pA, 2 s. **(C)** Representative traces of recorded mEPSCs and the boxplots of mEPSCs, showing similar mEPSC amplitude (Student's *t* test, *p* = 0.8525, *n* = 8 cells from 3 mice for each group) and frequency (Student's *t* test, *p* = 0.6440) in* Htr3a^-/-^* and WT mice. Scale bar: 20 pA, 2 s. **(D)** Representative traces of recorded mIPSCs and the boxoxplots of mEPSCs, showing similar mIPSC amplitude (Student's *t* test, *p* = 0.2205, *n* = 9 cells from 3 WT mice, *n* = 8 cells from 3 *Htr3a^-/-^* mice) and frequency (Mann Whitney test, *p* = 0.4234) in* Htr3a^-/-^* and WT mice. Scale bar: 20 pA, 2 s. Data are presented as boxplots (median and 5^th^-95^th^ percentile whiskers), or as mean ± SEM., * *p* < 0.05; ** *p* < 0.01, *** *p* < 0.001, **** *p* < 0.0001.

The hippocampus in human brain is crucial for memory 36, associated with autism 37, 38 and EP 39, suggesting that it may play an essential role in the behavioral phenotypes of the *Htr3a^-/-^* mice. The serotonergic system is involved in balancing E/I transmission 14-17. Therefore, we recorded the sEPSCs (**Figure 2A**) and sIPSCs (**Figure 2B**) on the pyramidal neurons in the hippocampal CA1 region by whole-cell voltage clamp. We observed no significant change in both frequency (Mann Whitney test, *p* = 0.7577) and average amplitude (Student's *t* test, *p* = 0.1108) of sEPSCs in the *Htr3a^-/-^* mice (**Figure 2A**). There was a higher frequency (Student's *t* test, *p* = 0.0422) of sIPSCs in *Htr3a^-/-^* mice, but no significant changes in amplitude (Student's *t* test, *p* = 0.1866) (**Figure 2B**). To assess the E/I balance of the same cell, we recorded the sEPSCs and sIPSCs on the pyramidal neurons at the holding voltage -70 mV and 0 mV, respectively (**Figure S3A-D**). Similar results for sEPSCs and sIPSCs were observed (**Figure S3B**, **Figure S3C**). The E/I ratio (**Figure S3D**) between the frequency of sEPSCs and sIPSCs was decreased, suggesting an enhanced inhibitor transmission (Student's *t* test, *p* = 0.0328). We blocked neuronal firing with the presence of 1 mM tetrodotoxin (TTX) and recorded miniature postsynaptic currents (**Figure 2C-D**). We found that in pyramidal cells of the hippocampal slices both mEPSCs and mIPSCs were not changed in* Htr3a* KO mice compared with WT (For mEPSC frequency, Student's *t* test, *p* = 0.6440; for mEPSC amplitude, Student's *t* test, *p* = 0.8525; for mIPSC frequency, Mann Whitney test, *p* = 0.4234; for mIPSC amplitude, Student's *t* test, *p* = 0.2205) in pyramidal cells of the hippocampal slices. These results suggested that the enhancement of sIPSCs frequency was dependent on the elevated excitability of the input GABAergic neurons.

### Differentially expressed genes in the hippocampus are enriched with genes involved in ASD, learning/memory and epilepsy

**Figure 3:**
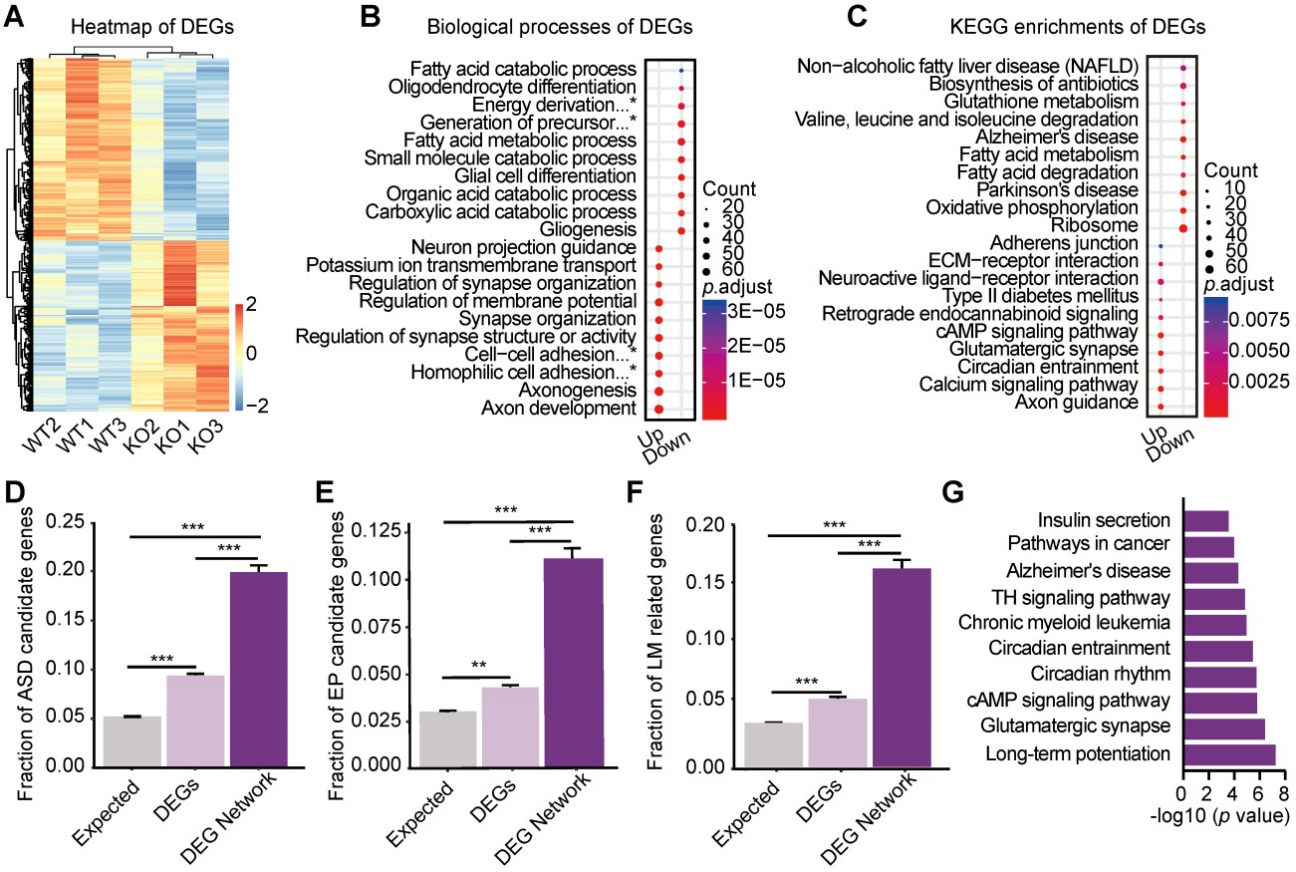
** Molecular networks involved in ASD, learning/memory, and epilepsy. (A)** Expression heatmap of the 2092 DEGs in the hippocampus. The regularized log2-transformed read counts were plotted using pheatmap package of DESeq2. Red color represents a relatively increased abundance, blue color represents a relatively decreased abundance, and white color represents no change. **(B)** Top 10 enriched Biological Processes in Gene Ontology (GO) of up-regulated genes and down-regulated genes (Table S3). *Complete terms: energy derivation by oxidation of organic compounds; generation of precursor metabolites and energy; cell-cell adhesion via plasma-membrane adhesion molecules; homophilic cell adhesion via plasma membrane adhesion molecules. **(C)** Top 10 enriched KEGG pathways of the up-regulated and down-regulated genes (Table S4). **(D)** Enrichment of ASD candidate genes in the DEG Network and DEGs. Fraction of ASD candidate genes = 5.34% (877/16,435) in the expressed genes; 9.42% (197/2,092) in DEGs; 20.41% (50/255) in the DEG Network (*p =* 2.0658E-16 between expressed genes and all DEGs; *p* = 1.2665E-16 between the expressed genes and the DEG Network, and *p* = 1.6545E-08 between all DEGs and the DEG Network). Error bars represent the standard error of the fraction, estimated using a bootstrapping method with 100 resamplings. Two-tailed Fisher's exact test, *** *p* < 0.001. **(E)** Enrichment of epilepsy (EP) candidate genes in the DEG Network and the DEGs. Fraction of EP candidate genes = 3.03% (498/16,435) in the expressed genes; 4.16% (87/2,092) in DEGs; 9.80% (24/245) in the DEG Network (*p =* 0.0021 between expressed genes and all DEGs; *p* = 4.5635E-07 between expressed genes and the DEG Network, and *p* = 4.3424E-05 between the DEGs and the DEG Network). Error bars represent the standard error of the fraction, estimated using a bootstrapping method with 100 resamplings. Two-tailed Fisher's exact test, ** *p* < 0.01, *** *p* < 0.001. **(F)** Enrichment of learning/memory (LM)-related genes in the DEG Network and the DEGs. Fraction of LM-related genes = 3.62% (596/16,435) in the expressed genes; 5.93% (124/2,092) in DEGs; 14.69% (36/245) in the DEG Network (*p =* 1.9173E-08 between expressed genes and all DEGs; *p* = 7.5313E-13 between expressed genes and the DEG Network, and *p* = 5.1941E-08 between the DEGs and the DEG Network). Error bars represent the standard error of the fraction, estimated using a bootstrapping method with 100 resamplings. Two-tailed Fisher's exact test, *** *p* < 0.001. **(G)** Top 10 enriched KEGG pathways of the DEG Network (*TH signaling pathway: thyroid hormone signaling pathway).

To further explore the underlying molecular mechanisms, we performed transcriptome profiling on 3 pools of hippocampi from 12 *Htr3a* KO mice and 3 pools from 12 control littermates. The samples of *Htr3a*^-/-^ mice were well separated from those of the littermate WT mice in the principal component analysis (PCA) (**Figure S4A**). Out of 16,435 expressed genes (**Table S1**), 2,092 were identified as differentially expressed genes (DEGs) (FDR ≤ 0.05) (**Figure 3A**), including 1,010 up-regulated and 1,082 down-regulated genes (**Figure S4B, Table S2**). The Gene Ontology (GO) (**Figure 3B and Figure S4C-D**, **Table S3**) biological processes, known to be associated with E/I balance and the regulation of neuronal excitability, such as axon development, axonogenesis, regulation of synapse structure or activity, synapse organization, and regulation of membrane potential, were found to be enriched. Many enriched KEGG (Kyoto Encyclopedia of Genes and Genomes) pathways (**Figure 3C**; **Table S4**) were also involved in the synaptic transmission and neuronal excitability, such as axon guidance, calcium signaling pathway, glutamatergic synapse, retrograde endocannabinoid signaling.

We constructed a mouse hippocampal interactome (**Table S5**) by integrating the hippocampal expressed genes with protein interaction data (See Methods). We then mapped the 2,092 differentially expressed genes (DEGs) to the mouse hippocampal interactome to retrieve the DEG Network (245 nodes and 231 edges) (**Figure S4E, Table S6**), including the DEGs and their first interacting neighbors with co-expression. The top major components of the DEG Network included four modules with distinct functions (named as ion channel regulation, neuronal transcription regulation, synaptic transmission and circadian rhythm regulation) (**Figure S4E, Table S7**), which are known to be related to autism 40-43.

Both DEGs and the DEG Network were enriched with ASD-candidate genes, LM-related genes, and EP-candidate genes, suggesting that DEG Network consisted of signaling pathways underlying the autistic-like phenotypes, impaired memory and reduced susceptibility to seizure. Compared with all DEGs, the DEG Network exhibited further enrichment with ASD candidate genes (**Table S8**) (two-tailed Fisher's exact test, ratio = 0.2041, *p* = 1.65E-08) (**Figure 3D**), EP candidate genes (**Table S9**) (two-tailed Fisher's exact test, ratio = 0.0980, *p =* 4.34E-05) (**Figure 3E**), and LM-related genes (**Table S10**) (two-tailed Fisher's exact test, ratio = 0.1469, *p* = 5.19E-08) (**Figure 3F**). The DEG Network was also enriched in signaling pathways involved in ASD, LM and EP (**Figure 3G**, **Table S11**), such as circadian rhythm 43, 44, cAMP pathway 45, 46, long-term potentiation 47, 48 and thyroid hormone signaling pathway 36, 49. Interestingly, many genes of the NMDAR system (*Grin1, Grin2a, Grin2b, Grin2c*) were involved in these pathways (**Figure 3G**).

### Upregulation of NMDAR pathways underlying ASD, learning/memory and epilepsy

**Figure 4:**
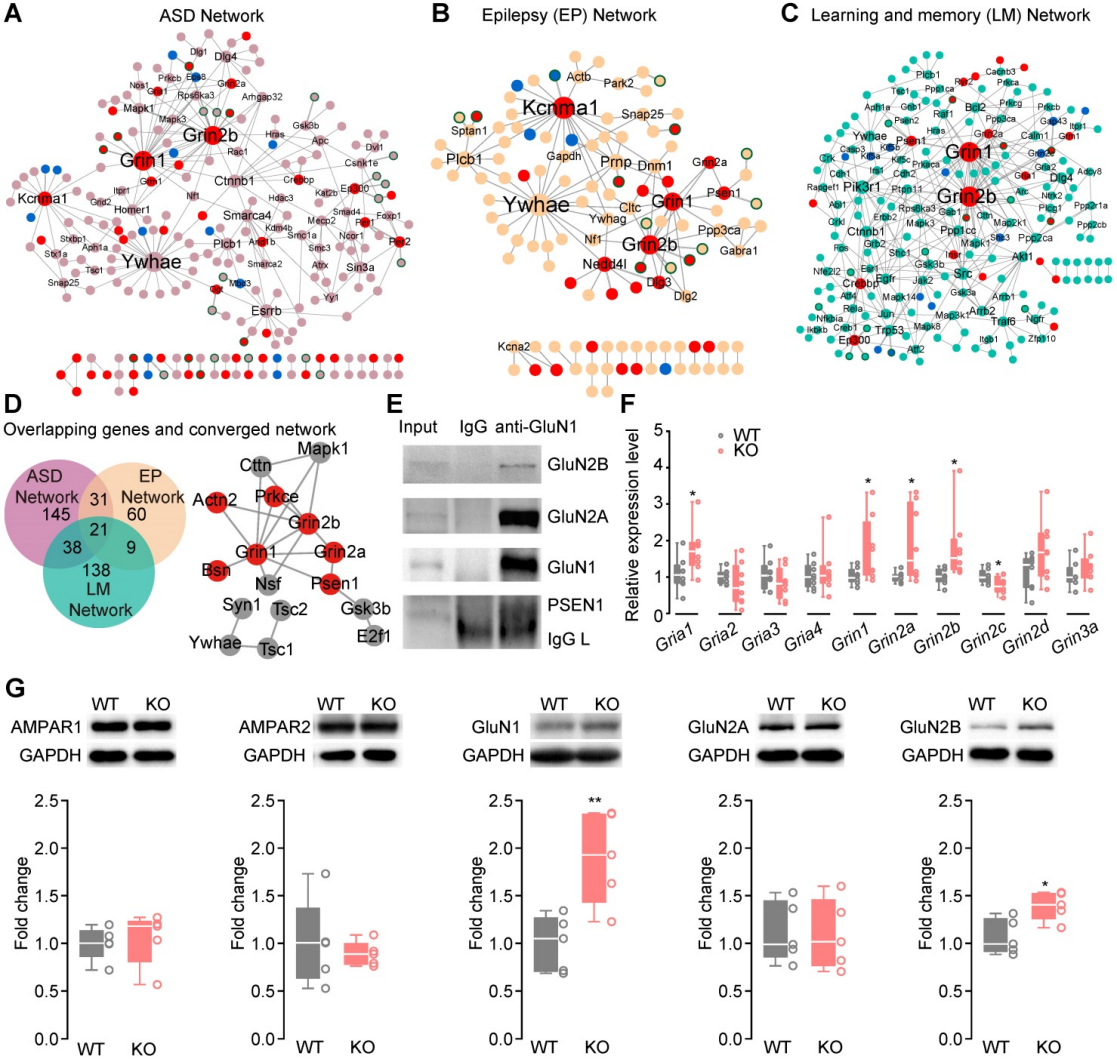
** Upregulation of NMDAR pathways underlying ASD, learning/memory and epilepsy. (A)** Protein interaction network for ASD (ASD Network). ASD candidate genes were mapped onto the hippocampal interactome to extract a network of ASD candidate genes and their first co-expressed neighbors. Most of the ASD candidate genes (light pink) show no expression changes. Red node: upregulated; blue node: downregulated; light pink node: without expression change; the node with green border: co-expressed neighbor; gray line: protein-protein interaction (PPI); double lines: PPI and co-expression; node size: degree; nodes with degree ≥ 3 are labeled in the panel. **(B)** Protein interaction network for epilepsy (EP Network). EP candidate genes were mapped onto the hippocampal interactome to extract a network consisting of the EP candidate genes and their first co-expressed neighbors. Most of the EP candidate genes (yellow) show no expression changes. Red node: upregulated; blue node: downregulated; yellow node: without expression change; node with green border: co-expressed neighbor; gray line: protein-protein interaction (PPI); double lines: PPI and co-expression; node size: degree; nodes with degree ≥ 3 are labeled in the panel. **(C)** Protein interaction network for learning and memory (LM Network). LM-related genes were mapped onto the hippocampal interactome to extract a network including the LM-related genes and their first co-expressed neighbors. Most of the LM-related genes (cyan) show no expression changes. Red node: upregulated; blue node: downregulated; cyan node: without expression change; node with green border: co-expressed neighbor; gray line: protein-protein interaction (PPI); double lines: PPI and co-expression; node size: degree; nodes with degree ≥ 3 are labeled in the panel. **(D)** Overlaps between the networks and the converged network module. There are 21 genes overlapping in the ASD-, EP and LM Networks. Red nodes: up-regulated genes, gray nodes: not changed genes. **(E)** Co-immunoprecipitation of GluN1 with GluN2B, GluN2A and PSEN1. **(F)** Relative expression levels of NMDAR and AMPAR genes in the hippocampus of *Htr3a^-/-^* mice were compared to WT mice (Student's* t* test, *p* = 0.1056 for *Gria1*, *p* = 0.3168 for *Gria2*, *p* = 0.6752 for* Gria3*, *p* = 0.6313 for *Gria4*, *p* = 0.0377 for *Grin1*, *p* = 0.0299 for *Grin2a*, *p* = 0.0360 for *Grin2b*, *p* = 0.0375 for *Grin2c*, *p* = 0.1066 for* Grin2d*, *p* = 0.3296 for* Grin3a*,* n* = 8 for each group). **(G)** Expression levels of NMDAR subunit GluN1 and GluN2B were increased in the hippocampus of *Htr3a^-/-^* mice compared with WT mice (Student's* t* test, *p* = 0.7277 for AMPAR1, *p* = 0.6083 for AMPAR2, *p* = 0.0076 for GluN1, *p* = 0.9121 for GluN2A, *p* = 0.0162 for GluN2B,* n* = 5 for each group). Data are presented as boxplots (median and 5^th^-95^th^ percentile whiskers), or mean ± SEM., * *p* < 0.05; ** *p* < 0.01, *** *p* < 0.001, **** *p* < 0.0001.

To further search for converged molecular pathways underlying ASD-, LM- and EP-related phenotypes, we mapped ASD candidate genes, LM-related genes or EP candidate genes to the mouse hippocampal interactome network respectively, and retrieved three subnetworks (ASD Network, EP Network and LM Network). The ASD Network (**Figure 4A**, **Table S12**), LM Network (**Figure 4B, Table S13**) and EP Network (**Figure 4C, Table S14**) included their candidate genes and their first co-expressed protein-protein interaction (PPI) neighbors. Of the genes in the ASD-, LM- and EP networks, 26.38% (62/235), 16.99% (35/206), 24.79% (30/121) were DEGs.

The upregulation of NMDAR subunit genes *Grin2b and Grin1* were among the top hub genes in the ASD- (**Table S15**), EP- (**Table S16**) and LM (**Table S17**) Networks. Of the 143 enriched KEGG pathways of the ASD-, EP- and LM Networks (**Figure S5, Table S18** ), 16 pathways contain NMDAR. Interestingly, 87.5% (14/16) of the NMDAR-containing pathways were shared by all three networks (**Table S12**-**S14**). The serotonergic synapse, glutamatergic synapse, GABAergic synapse pathways were shared by the ASD-, EP- and LM Networks. These results were consistent with previous findings that the serotonergic, GABAergic and glutamatergic systems are commonly involved in autism 4, 5.

The ASD, EP, and LM networks with 21 common genes in a shared network module included proteins encoded by *Grin1*, *Grin2a,* and *Grin2b* and their interactors (**Figure 4D**), suggesting the involvement of the common NMDAR system in the three phenotypic domains in *Htr3a^-/-^* mice. We performed immunoprecipitation of the endogenous proteins in the mouse hippocampus to confirm GluN1-GluN2A, GluN1-GluN2B and GluN1-PSEN1 interactions (**Figure 4E**). The expression of NMDAR and AMPAR was quantified by qRT-PCR (**Figure 4F, Table S19**) and Western blotting (**Figure 4G**) to verify the upregulated expression of the gene products. The expression of GluN1 and GluN2B subunits was higher in the hippocampus of the KO mice. Since the action potential-driven GABAergic input in pyramidal neurons was enhanced and glutamatergic and GABAergic synapse pathways were shared by ASD, EP, and LM networks (**Figure S5**), we speculated that the elevated expression of NMDAR genes might upregulate the excitability of GABAergic neurons.

### Increased NMDAR function in PV^+^ interneurons

Both cholecystokinin positive (CCK^+^)-pyramidal neuron inhibitory and CCK^+^-PV^+^-pyramidal neuron disinhibitory connections 50 control the GABAergic input of pyramidal neurons in the hippocampus. In the GABAergic synapse pathway shared by the ASD-, EP- and LM Networks, *Cacna1a* and *Nsf* were upregulated (**Figure S5, Table S18**). The *Cacna1a* gene encodes the P/Q calcium channel, which is specifically expressed in the PV^+^ interneuron and controls the GABA release in the hippocampus 50. NSF (N-ethylmaleimide sensitive factor), an ATPase associated with various cellular activities protein, is required for intracellular membrane fusion. Notably, it is reported that NSF is required for the NMDAR-potentiated inhibitory transmission 51. Since *Cacna1a* is uniquely expressed in PV^+^ interneurons, the upregulation of *Cacna1a* indicated that it might be involved in the increased GABA release in PV^+^ interneurons. We applied P/Q calcium channel antagonist, ω-agatoxin-TK, in the sIPSCs recording. ω-agatoxin-TK (0.25 μM) decreased both the frequency and amplitude of sIPSC, indicating that the P/Q calcium channel-dependent GABA release may be the source of enhanced GABAergic input in the *Htr3a^-/-^* mice (**Figure S6A-C**).

**Figure 5:**
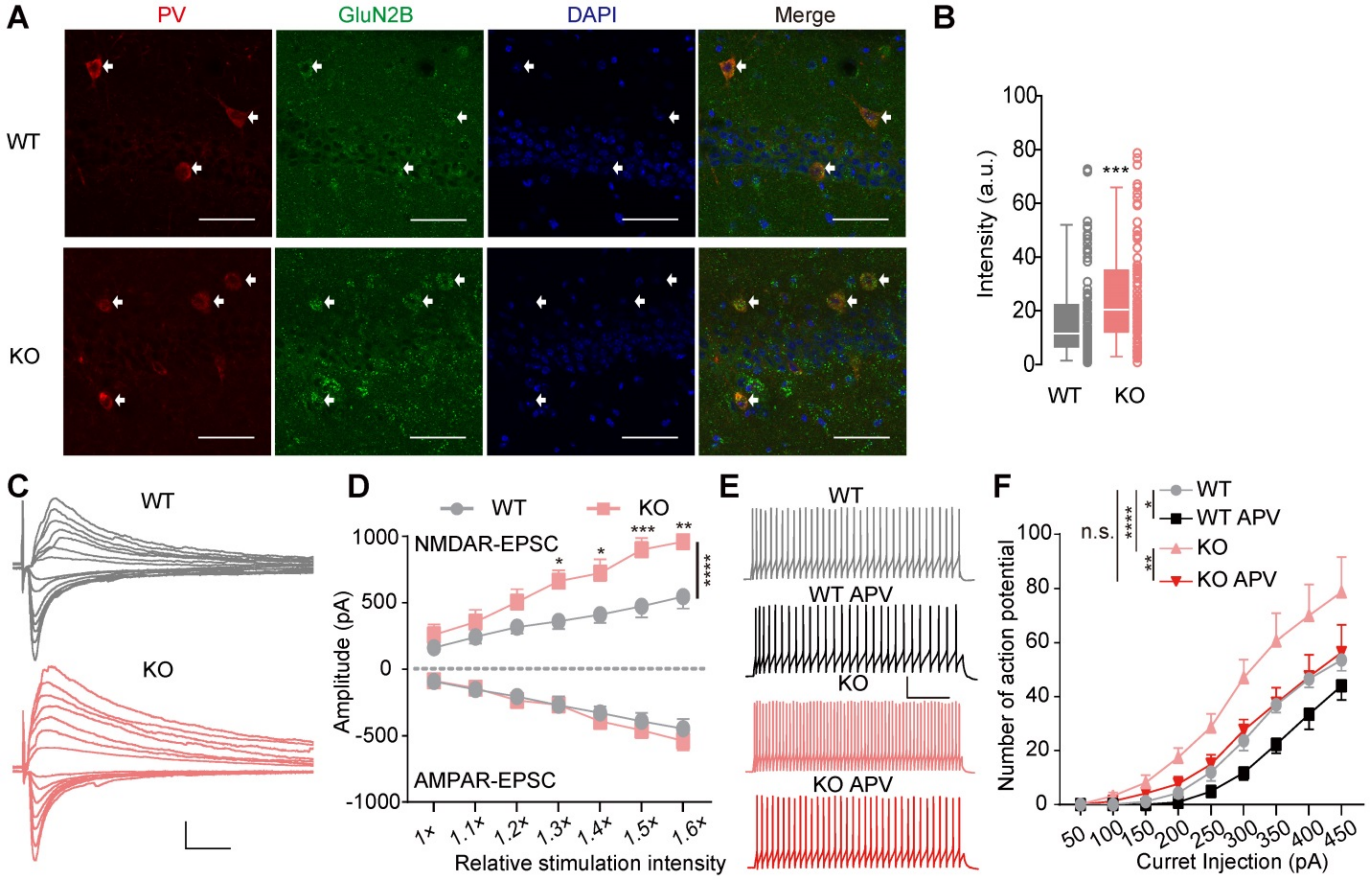
** Increased excitability of PV+ interneurons in *Htr3a^-/-^* mice by NMDAR upregulation. (A)** Significant increase in GluN2B immunoreactivity (green) in the PV^+^ (red) neurons in the CA1 region of *Htr3a^-/-^* mice (bottom row), compared with controls (top row). Scale bar: 50 µm. **(B)** Quantified data showing a significant increase of GluN2B in PV^+^ neurons in the CA1 region of *Htr3a^-/-^* mice. (Student's* t* test, *p* = 0.0005, *n* = 93 cells from 3 WT mice, *n* = 100 cells from 3 KO mice). **(C)** Representative traces of evoked AMPAR- (held at -70 mV) and NMDAR-current (held at +40 mV) in PV^+^ neurons in the CA1 region of *Htr3a^-/-^* and WT mice. Scale bar: 20 pA, 100 ms. **(D)** Normal AMPAR current but increased NMDAR current in PV^+^ neurons in the CA1 region of *Htr3a^-/-^* mice. (For AMPAR current, two-way ANOVA, F_1, 140_ = 1.831, *p* = 0.0001; for NMDAR current, two-way ANOVA, F_1, 140_ = 31.27, *p* < 0.0001. *n* = 13 cells from 3 WT mice, *n* = 9 cells from 3 KO mice). **(E)** Voltage traces evoked by 300 pA current injection before and after D-APV (50 µM) treatment of *Htr3a^-/-^* and WT mice. Scale bars represent 100 ms, 20 Mv. **(F)** Firing number plotted against depolarizing currents. (*n* = 9 cells from 3 WT mice, *n* = 12 cells from 3 KO mice; two-way ANOVA, WT vs. KO, F_1,171_= 25.95, *p* < 0.0001; two-way ANOVA by matching factors, WT vs. WT APV, F_1,8_= 9.451, *p* = 0.0152; two-way ANOVA by matching factors, KO vs. KO APV, F_1,11_= 10.60, *p* = 0.0077; two-way ANOVA, WT vs. KO APV, F_1,19_= 0.3283, *p* = 0.5734). Data are presented as boxplots (median and 5^th^-95^th^ percentile whiskers), or as mean ± SEM., * *p* < 0.05; ** *p* < 0.01, *** *p* < 0.001, **** *p* < 0.0001.

Next, we immune-stained hippocampal slices for GluN2B and PV and observed GluN2B upregulation in CA1 PV^+^ interneurons in *Htr3a^-/-^* mice (Mann Whitney test, *p =* 0.0005, **Figure 5A-B**). We crossed PV-Cre:Ai14 mice and *Htr3a^-/-^* mice to mark PV^+^ cells in *Htr3a^-/-^* mice, and recorded the NMDAR and AMPAR input-output curve on PV^+^ interneurons (**Figure 5C**). The NMDAR current of PV^+^ interneurons was larger in the PV-Cre:Ai14:*Htr3a^-/-^* mice than the PV-Cre:Ai14 mice (two-way ANOVA, *p* < 0.0001), while the AMPAR current was unchanged (two-way ANOVA, *p* = 0.1782) (**Figure 5D**). We also found the NMDAR current to be larger in hippocampal pyramidal neurons in KO mice than the controls (two-way ANOVA, *p* < 0.0001), while the AMPAR current was normal in pyramidal neurons in the *Htr3a^-/-^* mice (two-way ANOVA, *p* = 0.6975) (**Figure S6D-F**).

To determine whether the enhanced of NMDAR current regulated the excitability of PV^+^ interneurons, we evaluate the spike number of PV^+^ interneurons by the injection of depolarizing current steps (500 ms) to characterized the firing properties (**Figure 5E**). Compared to the controls, the excitability of hippocampal PV^+^ interneurons was increased in PV-Cre:Ai14:*Htr3a^-/-^* mice (two-way ANOVA, *p* < 0.0001) (**Figure 5F**). These results were consistent with the enhanced presynaptic action potential-driven GABAergic input of pyramidal neurons in the *Htr3a^-/-^* mice (**Figure 2**). Moreover, the D-APV, an antagonist of NMDAR, decreased the firing frequency of PV^+^ interneurons in both WT (two-way ANOVA, *p* = 0.0152) and KO mice (two-way ANOVA, *p* = 0.0077) (**Figure 5F**). Most importantly, D-APV application reduced the excitability in the KO mice to a level similar to that of WT mice (two-way ANOVA, *p* = 0.5734), suggesting that the enhanced excitability of PV^+^ interneurons in the KO mice was dependent on the upregulation of NMDAR, and contributed to the enhanced inhibitory GABAergic transmission and decreased E/I ratio in pyramidal neurons.

### Autistic-like deficits in the *Htr3a*^-/-^ mice rescued by NMDAR blockade

**Figure 6:**
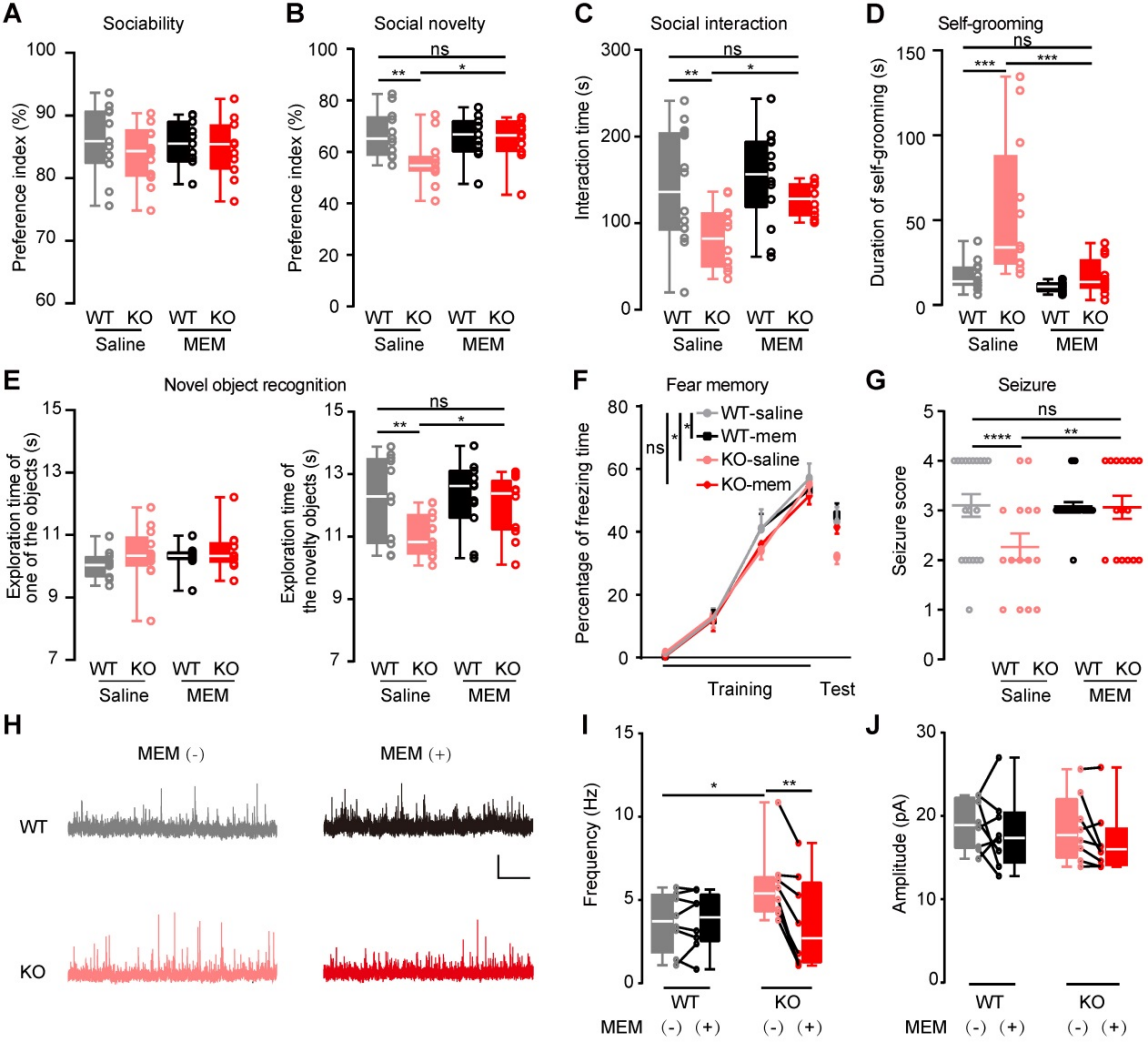
** Rescue of autistic-like deficits in* Htr3a^-/-^* mice by NMDAR blockade.** The effect of memantine (MEM) on mouse behaviors was tested (A-G) by i.p. administered memantine (5 mg/kg) or saline alone (control) to *Htr3a^-/-^* mice or WT mice 30 min before behavior tests. The effect of memantine on neuronal transmission was tested (H-J) by electrophysiological recordings in a bath with (+)/without (-) 1 µM memantine. **(A)** Effect of memantine on sociability. In the 10-min sociability phase of social approach task, the social preference index of interaction with a stranger mouse was determined. Treatment of memantine had no effect on sociability of both *Htr3a^-/-^* and WT mice (two-way ANOVA, F_1,43_ = 1.135, *p* = 0.2927. *p* = 0.2659 between saline-treated *Htr3a^-/-^* and WT mice, *p* = 0.8229 between memantine-treated and saline-treated WT mice, *p* > 0.9999 between memantine-treated and saline-treated *Htr3a^-/-^* mice,* p* = 0.5347 between memantine-treated *Htr3a^-/-^* and saline-treated WT mice; *n* = 11 for saline-treated WT mice; *n* = 14 for saline-treated *Htr3a^-/-^* mice, *n* = 11 for memantine-treated WT mice, *n* = 11 for memantine-treated *Htr3a^-/-^* mice). **(B)** Effect of memantine on social novelty. In the 10-minute social novelty phase of social approach task, memantine treatment significantly rescued the social preference of *Htr3a^-/-^* mice to interact with a stranger mouse over a familiar mouse (two-way ANOVA, F_1,43_ = 5.655, *p* = 0.0219. *p* = 0.0029 between saline-treated *Htr3a^-/-^* and saline-treated WT mice, *p* = 0.7603 between memantine-treated and saline-treated WT mice, *p* = 0.0153 between memantine-treated *Htr3a^-/-^* and saline-treated *Htr3a^-/-^* mice,* p* = 0.5541 between memantine-treated *Htr3a^-/-^* and saline-treated WT mice; *n* = 11 for saline-treated WT mice; *n* = 11 for saline-treated WT mice, *n* = 14 for saline-treated *Htr3a^-/-^* mice, *n* = 11 for memantine-treated WT mice, *n* = 11 for memantine-treated *Htr3a^-/-^* mice). **(C)** Effect of memantine on social interaction. Quantification of the results was based on the time of active interaction in the 10-min social interaction tests. Memantine treatment significantly rescued active social interactions of *Htr3a^-/-^* mice compared to treatment with saline (two-way ANOVA, F_1, 46_ = 8.790, *p* = 0.0048. *p* = 0.0040 between saline-treated *Htr3a^-/-^* and WT mice, *p* = 0.5802 between memantine-treated and saline-treated WT mice, *p* = 0.0412 between memantine-treated and saline-treated *Htr3a^-/-^* mice,* p* = 0.4847 between memantine-treated *Htr3a^-/-^* and saline-treated WT mice; *n* = 11 for saline-treated WT mice; *n* = 14 for saline-treated WT mice, *n* = 12 for saline-treated *Htr3a^-/-^* mice, *n* = 14 for memantine-treated WT mice, *n* = 10 for memantine-treated *Htr3a^-/-^* mice). **(D)** Memantine treatment significantly reduced repetitive self-grooming activity in the *Htr3a^-/-^* mice (two-way ANOVA, F_1, 44_ = 11.67, *p* = 0.0014. *p* = 0.0009 between saline-treated *Htr3a^-/-^* WT mice, *p* > 0.9999 between saline-treated WT and memantine-treated WT mice, *p* = 0.0007 between memantine-treated *Htr3a^-/-^* and saline-treated *Htr3a^-/-^* mice, *p* > 0.9999 between memantine-treated *Htr3a^-/-^* and saline-treated WT mice; *n* = 14 for saline-treated WT mice, *n* = 12 for saline-treated *Htr3a^-/-^* mice, *n* = 14 for memantine-treated WT mice, *n* = 12 for memantine-treated *Htr3a^-/-^* mice). **(E)** Effect of memantine on novel object recognition. Memantine ameliorated novel object recognition deficit in the *Htr3a^-/-^* mice (for familiarization phase (left panel), two-way ANOVA, F_1,43_ = 1.7780, *p* = 0.1894. *p* = 0.2316 between saline-treated *Htr3a^-/-^* and WT mice, *p* = 0.3828 between memantine-treated WT and saline-treated WT mice, *p* = 0.6883 between memantine-treated and saline-treated *Htr3a^-/-^* mice, *p* = 0.1219 between memantine-treated *Htr3a^-/-^* and saline-treated WT mice; for test phase (right panel), two-way ANOVA, F_1,43_ = 5.918, *p* = 0.0192. *p* = 0.0091 between saline-treated *Htr3a^-/-^* and WT mice, *p* = 0.9182 between memantine-treated and saline-treated WT mice, *p* = 0.0371 between memantine-treated and saline-treated *Htr3a^-/-^* mice, *p* = 0.5385 between memantine-treated *Htr3a^-/-^* and saline-treated WT mice; *n* = 11 for saline-treated WT mice, *n* = 13 for saline-treated *Htr3a^-/-^* mice, *n* = 11 for memantine-treated WT mice, *n* = 12 for memantine-treated *Htr3a^-/-^* mice). **(F)** Effect of memantine on fear memory. In the contextual fear conditioning test, 24 h after 4 times of tone-shock paired training, the percentage of freezing time during 2 min of contextual fear test was compared. Memantine treatment rescued performance deficits of *Htr3a^-/-^* mice in fear memory (two-way ANOVA, F_1,43_ = 5.309, *p* = 0.0261. *p* = 0.0178 between saline-treated *Htr3a^-/-^* and WT mice, *p* = 0.7052 between memantine-treated and saline-treated WT mice, *p* = 0.0434 between memantine-treated and saline-treated *Htr3a^-/-^* mice, *p* = 0.6754 between memantine-treated *Htr3a^-/-^* and saline-treated WT mice; *n* = 11 for saline-treated WT mice, *n* = 13 for saline-treated *Htr3a^-/-^* mice, *n* = 11 for memantine-treated WT mice, *n* = 12 for memantine-treated *Htr3a^-/-^* mice). **(G)** Effect of memantine on the susceptibility to PTZ-induced seizures. *Htr3a^-/-^* mice treated with memantine showed significantly increased seizure scores after PTZ injection (two-way ANOVA, F_1,59_ = 21.39, *p* < 0.0001. *p* < 0.0001 between saline-treated *Htr3a^-/-^* and WT mice, *p* = 0.3784 between memantine-treated and saline-treated WT mice, *p* = 0.0022 between memantine-treated and saline-treated *Htr3a^-/-^* mice, *p* = 0.0629 between memantine-treated *Htr3a^-/-^* and saline-treated WT mice; *n* = 20 for saline-treated WT mice, *n* = 15 for saline-treated *Htr3a^-/-^* mice, *n* = 24 for memantine-treated WT mice, *n* = 16 for memantine-treated *Htr3a^-/-^* mice). **(H-J)** Memantine reduced sIPSC frequency to a normal level in *Htr3a^-/-^* mice. Representative traces of sIPSCs recorded in *Htr3a^-/-^* and WT mice, scale bar, 20 pA, 2 s (H). Memantine significantly decreased sIPSC frequency (J) but not amplitude (I) in the *Htr3a^-/-^* mice (for frequency, WT_(-)_ vs. KO_(-)_, Student's *t* test* p* = 0.0241, WT_(-)_ vs. WT_(+)_, Paired *t* test* p* = 0.3411, KO_(-)_ vs. KO_(+)_, Paired *t* test* p =* 0.0013, WT_(-)_ vs. KO_(+)_, Student's *t* test *p* = 0.8583; for amplitude, WT_(-)_ vs. KO_(-)_, Student's *t* test* p* = 0.8086, WT_(-)_ vs. WT_(+)_, Paired *t* test* p* = 0.5556, KO_(-)_ vs. KO_(+)_, Paired *t* test* p =* 0.1230; *n* = 8 cells from 3 WT mice, *n* = 8 cells from 4 *Htr3a^-/-^* mice). Data are presented as boxplots (median and 5^th^-95^th^ percentile whiskers), or as mean ± SEM., * *p* < 0.05; ** *p* < 0.01, *** *p* < 0.001, **** *p* < 0.0001.

To verify the upregulated NMDAR in autistic-like behaviors, memantine (5 mg/kg) was i.p. administrated to mice to inhibit NMDAR. The saline-treated *Htr3a*^-/-^ mice displayed the same deficits as described above (**Figure 1**). The injected memantine in *Htr3a^-/-^* and WT mice did not affect their social ability (**Figure 6A**). In the social novelty phase, memantine-treated *Htr3a^-/-^* mice spent significantly more time with the stranger 2 mouse than the familiar one (stranger 1) compared to saline-treated *Htr3a^-/-^* mice (two-way ANOVA, *p* = 0.0153, **Figure 6B**), to a level comparable to the wild type control (two-way ANOVA,* p* = 0.5541, **Figure 6B**). In the home-cage social interaction test, memantine could significantly increase the time in active interactions, including allo-grooming, following and mounting, in the *Htr3a^-/-^* mice (two-way ANOVA, *p =* 0.0412, **Figure 6C**), while memantine-treated KO mice showed a similar level of active interactions as saline-treated WT mice (two-way ANOVA,* p* = 0.4847, **Figure 6C**). Acute memantine administration also significantly rescued repetitive self-grooming in the *Htr3a^-/-^* mice (two-way ANOVA, *p =* 0.0007, **Figure 6D**), to a level similar to saline-treated WT mice (two-way ANOVA, *p* > 0.9999) (**Figure 6D**). We also observed that memantine administration could significantly increase the exploration time of the novel object in the *Htr3a*^-/-^ mice (two-way ANOVA, *p* = 0.0371, **Figure 6E**), to a level comparable to wild type mice (two-way ANOVA, between saline-treated WT and memantine-treated KO mice,* p* = 0.5385, **Figure 6E**). In contextual fear conditioning test, we found that memantine treatment significantly increased fear memory of *Htr3a*^-/-^ mice (two-way ANOVA, *p* = 0.0434, **Figure 6F**), to a level comparable to the WT control animals (two-way ANOVA, between saline-WT and memantine-treated KO mice,* p* = 0.6754, **Figure 6F**). Also, the *Htr3a*^-/-^ mice treated with memantine showed increased PTZ-induced seizure scores (two-way ANOVA, between saline- and memantine-treated KO mice, *p* = 0.0022; between saline-WT and memantine-treated KO mice,* p* = 0.0629) (**Figure 6G**). However, memantine did not affect the behaviors of WT mice (**Figure 6A-G**). These results demonstrated that inhibition of NMDAR with memantine could rescue these behavioral phenotypes of the *Htr3a*^-/-^ mice.

Since we observed increased frequency of sIPSCs in the *Htr3a*^-/-^ mice (**Figure 2**), suggesting enhanced inhibitory drive to pyramidal neurons, we further assessed memantine effects on the inhibitory input to pyramidal neurons in the *Htr3a*^-/-^ mice. We used 1 μM memantine, which is within the therapeutic brain concentration range (~0.5-1 μM) 52. Memantine significantly reduced sIPSC frequency but not amplitude in the *Htr3a*^-/-^ mice (paired *t* test, *p* = 0.0013 for frequency in KO mice bath with or without memantine; *p* = 0.1230 for amplitude in KO mice bath with or without memantine), while it did not affect sIPSCs in WT mice (**Figure 6H-J**). These results of memantine-rescue experiments, together with the findings on upregulation of NMDAR and enhanced excitability in the PV^+^ interneurons (**Figure 5**), provided evidence that increasing the NMDAR signaling in PV^+^ interneurons enhanced GABAergic transmission and thereby caused the imbalance of E/I leading to the autistic-like behaviors.

## Discussion

The *Htr3a^-/-^* mice displayed autistic-like behaviors (**Figure 1**, **Figure S2**), including less active social interactions, impaired social novelty, decreased social odor interest and increased repetitive selfgrooming. In a previous study, *Htr3a* knockout mice displayed impaired sociability but with normal social interactions 32, while the *Htr3a^-/-^* mice showed normal sociability (**Figure 1A, Figure S2A**) but less active interactions in the home cage social interaction test (**Figure 1C, Figure S2C**). Such discrepancy could be due to experimental settings: the previous study used new cages in the social interaction tests 32, while we used home cages to minimize environmental stress. The social interaction test, which allows direct contact with stranger animals within the home cages, may be more sensitive to the detection of social deficits in rodents 53, 54.

The olfactory habituation/dishabituation test suggested that the *Htr3a^-/-^* mice showed intact olfaction, but a decreased interest in the social stimulus odors (**Figure 1D; Figure S2D**), which may be interpreted as decreased interest in social interactions. Apart from the core autistic behaviors, the *Htr3a^-/-^* mice showed impaired memory (**Figure 1F-G; Figure S2H-I**), consistent with the reduced IQ in most of the autistic children 3. The *Htr3a^-/-^* mice also showed attenuated susceptibility to seizures (**Figure 1H**), which is the antithesis of the comorbidity between ASD and EP in humans 55. The potential mechanism of attenuated susceptibility to seizures in the *Htr3a* KO mice might be due to the decreased E/I ratio by NMDAR upregulation in the PV^+^ interneurons. Previous studies performed in ASD patients and mouse models indicated that the E/I imbalance toward hyperexcitability might contribute to ASD-EP comorbidity 55. Both increased 12 and decreased 13 the E/I ratio were observed in the brains of autistic mouse models, suggesting that the E/I imbalance, either decreased or increased E/I ratio, may underly autism 56.

The hippocampal DEGs are enriched with ASD candidate genes, LM-related genes and EP candidate genes. It is of note that the DEG Network showed further enrichment with these groups of genes (**Figure 3D-F**), suggesting that the DEGs are involved in the major pathways underlying the three phenotypes. The major components of the DEG Network included subnetworks enriched for functions such as ion channel regulation, transcription regulation, synaptic transmission and circadian rhythm regulation (module 1-4 in **Figure S4E**). We searched the literature for every node in the synaptic transmission module (module 3) (**Figure S4E**), and found that 50% (14/28) of the genes were involved in ASD, 28.5% (8/28) of the genes are involved in EP, and 21.4% (6/28) of the genes are involved in both ASD and EP (**Table S7**). In this network module, *Grin1*, *Grin2a* and *Grin2b,* encoding for subunits of the NMDAR, were both hub and up-regulated genes. NMDAR are known to be the key regulators in the molecular pathways in learning and memory 57. Previous studies have suggested that *GRIN1, GRIN2A,* and* GRIN2B* were associated with autism 58 and epileptic disorders 59, 60. These results led us to further search for converged pathways involved in the three phenotypic domains.

The ASD Network, LM Network and EP Network contain up-regulated *Grin1* and *Grin2b*, which are also hub genes of these networks (**Figure 4D**), suggesting that NMDAR-related pathways might be the common pathways underlying autistic-like behaviors, impaired learning/memory and attenuated susceptibility to seizure. These results are consistent with previous findings of the NMDAR system. Some mutations in *GRIN1* were associated with intellectual disability, behavioral abnormalities, and stereotypical movements 61, while others in *GRIN2A* correlated with a spectrum of neurodevelopmental disorders with speech delay, apraxia and EP 62; similarly, some mutations in *GRIN2B* were associated with neurodevelopmental disorders characterized by mild to profound developmental delay/intellectual disability, often with EP and ASD behaviors 63.

Some functions (GO terms and KEGG pathways) associated with E/I balance and the regulation of action potential were enriched in DEGs and the DEG Network (**Figure 3**). The GABAergic input to pyramidal neurons mainly comes from CCK^+^ and PV^+^ neurons in the hippocampus 64. We found upregulation of *Cacna1a* (encoding the P/Q-type calcium channel), which is one of key shared genes in the converged pathways (**Figure S6**, **Table S18**). It has been reported that the P/Q-type calcium channel is specifically expressed in PV^+^ neurons, and thus exclusively mediates GABA release from hippocampal PV^+^ interneurons 50. We found that the P/Q-type calcium channel antagonist, ω-agatoxin-TK, rescued enhanced sIPSCs in the KO mice, suggesting the key role of PV^+^ interneurons in the GABAergic input to pyramidal neurons. We observed increased NMDAR expression and enhanced NMDAR-dependent excitability in the hippocampal PV^+^ neurons in the *Htr3a*^-/-^ mice (**Figure 5**), consistent with the increased action potential-dependent GABAergic transmission detected in pyramidal neurons (**Figure 2**). The NMDAR antagonist D-APV rescued the excitability of PV^+^ neurons and memantine rescued sIPSCs, indicating that the NMDAR system in PV^+^ neurons may play a critical role in GABAergic transmission and E/I balance. The NMDAR antagonist memantine rescued autistic-like features, impaired memory, attenuated seizure susceptibility in *Htr3a^-/-^* mice (**Figure 6A-G**), and that memantine suppressed the GABAergic transmission of pyramidal neurons in the *Htr3a^-/-^* mice (**Figure 6H-J**). These results also suggest that the NMDAR system can be targeted in designing therapy for autism. Since the NMDAR antagonist memantine is used in adjunct therapy for autism 65, 66, other proteins in the NMDAR pathways can be potential therapeutic targets.

Taken together, the *Htr3a* KO mice exhibited autistic-like behaviors, impaired learning/memory and attenuated PTZ-induced seizures, suggesting that *HTR3A* as a potential causal gene of autism. The transcriptome sequencing of the mouse hippocampus revealed a landscape of dysregulated genes with significant enrichment of ASD, EP candidate genes and LM related genes. Analysis of the converged molecular networks suggested a key role for NMDAR in PV^+^ interneurons in determining the phenotypes, which was further confirmed by the rescue experiments in the KO mice using the NMDAR antagonist memantine.

## Supplementary Material

Supplementary methods, figures and table legends.

Supplementary tables.

## Abbreviations

AMPAR: α-amino-3-hydroxy-5-methyl-4-isoxazole-propionic acid receptor
ANOVA: Analysis of variance
ASD: Autism spectrum disorder
BioGRID: Biological general repository for interaction datasets
cAMP: Cyclic Adenosine monophosphate
CCK^+^: cholecystokinin positive
DAVID: Database for annotation, visualization and integrated discovery
DEG: Differentially expressed gene
EP: Epilepsy
EPM: Elevated plus maze
E/I: excitatory/inhibitory
FDR: False discovery rate
FPKM: Fragments per kilobase of transcript per million mapped reads
GABA: γ-aminobutyric acid
GO: Gene ontology
5-HT3: serotonin 3 receptor
IQ: Intelligence quotient
KEGG: Kyoto Encyclopedia of Genes and Genomes
KO: knockout
LM: Learning/memory
MEM: memantine
mIPSCs: miniature inhibitory postsynaptic currents
nACh: nicotinic acetylcholine
NOR: novel object recognition
NMDAR: N-methyl-D-aspartate receptor
OFT: Open field test
PV: parvalbumin
PCA: Principle Components Analysis
PPI: Protein-protein interaction
PTZ: Pentylenetetrazol
PV^+^: parvalbumin positive
RIN: RNA integrity number
SEM: errors of the means
sEPSCs: spontaneous excitatory postsynaptic currents
sIPSCs: spontaneous inhibitory postsynaptic currents
TALEN: Transcription activator-like (TAL) effector nucleases
WT: Wild type
