# Supplementary material for: Upregulated NMDAR-mediated GABAergic transmission underlies autistic-like deficits in Htr3a knockout mice
Source: Theranostics. 2021 Sep 7;11(19):9296–310. doi: 10.7150/thno.60531 (PMC8490518; doi:10.7150/thno.60531)
Supplement: Supplementary file 1 — Supplementary methods, figures and table legends. [file thnov11p9296s1.pdf]

Supplemental Information

Upregulated NMDAR-mediated GABAergic transmission underlies autistic-like deficits in *Htr3a* knockout mice

TABLE OF CONTENTS

Methods

Behavior tests

1. Animal housing and handling
2. Social approach task
3. Home cage social interaction test
4. Olfactory habituation/dishabituation test
5. Self-grooming test
6. Novel object recognition
7. Contextual fear conditioning
8. Elevated plus maze test
9. Open field test
10. Seizure susceptibility test
11. Memantine treatment

RNA-seq and differential expression analysis

Functional enrichment analyses for differentially expressed genes

1. Functional annotations of up-regulated and down-regulated genes
2. Data source for genes used for enrichment analyses

Construction of hippocampal interactome and DEG interactome

Networks for autism, epilepsy and learning/memory

Figures

Figure S1. Genotyping and behavioral deficits.

Figure S2. Female *Htr3a* KO mice exhibited impaired social behavior and memory.

Figure S3. Enhanced GABAergic transmission perturbed excitatory/inhibitory balance.

Figure S4. Transcriptome sequencing and DEG Network.

Figure S5. Comparison of enriched pathways with ASD-, EP- and LM Networks.

Figure S6. The P/Q calcium channel mediated the GABAergic transmission and the NMDAR current were enhanced in the pyramidal neurons in the CA1 region of *Htr3a*$^{-/-}$ mice.

Tables (An excel table containing 19 sheets is uploaded separately)

Table S1. The 16,435 expressed genes list.

Table S2. The 2,092 differentially expressed genes list.

Table S3. Enrichment analysis for gene ontology (GO) annotations involved in DEGs.

Table S4. The KEGG clusters involved in DEGs.

Table S5. The genes list of hippocampal interactome.

Table S6. The genes list of DEG Network.

Table S7. The nodes information list of hippocampal differential interactome network.

Table S8. The list of 1,036 ASD candidate genes.

Table S9. The list of 647 epilepsy candidate genes.

Table S10. The list of 909 learning/memory related genes.

Table S11. The KEGG clusters involved in DEG Network.

Table S12. The genes and edges list of ASD Network.

Table S13. The genes and edges list of epilepsy Network.

Table S14. The genes and edges list of LM Network.

Table S15. The top 10 hub genes in ASD Network.

Table S16. The top 10 hub genes in epilepsy Network.

Table S17. The top 10 hub genes in LM Network.

Table S18. The common KEGG clusters involved in three networks.

Table S19. The list of the primers used for mRNA quantification.

Methods

Behavior tests

1. Animal housing and handling. Homozygous *Htr3a* knockout mice (*Htr3a*$^{-/-}$) and WT littermate controls were generated by breeding heterozygous mice. Mice were group housed (4-5 mice per cage) under Specific Pathogen Free (SPF) conditions, given a 12h:12h light-dark cycle and allowed *ad libitum* access to food and water. Before behavioral tests, mice were handled for 3 days and taken into the testing rooms 30-60 minutes.

2. Social approach task. The procedure for social approach task was slightly modified from the method described previously [1]. Specifically, the testing apparatus was a rectangular clear Plexiglas three chambers box (60 cm (L) x 40 cm (W) x 20 cm (H)). The dividing walls had doorways allowing mouse access to each chamber. The stranger mice from the same strain were habituated to placement inside the wire cage for 5 days prior to testing. Each test mouse was first placed into the center chamber with open access to both left and right chamber, each chamber containing an empty round wire cage. The wire cage (12 cm (H), 11 cm diameter) allows nose contact between mice but prevents fighting. After 10 min of habituation, during the social phase, an age-matched stranger was placed in the one wire cage while the opposite one is empty. The test mouse was allowed to freely explore the social apparatus for 10 min to test whether it prefers to interact with the object (O) or the stranger mouse (S1). Sniffing time was plotted as a social preference index = $T_{S1}/(T_{S1}+T_O)$, T_{S1} – time for a testing mouse interacting with a novel mouse (S1, Stranger1), T_O – time for a testing interacting with an empty cage (O, Object). To evaluate the preference for a novel stranger, the test mouse was then tested in a second 10-min session, which contains a novel stranger (S2) in the opposite wire cage. Sniffing time was plotted as a social preference index = $T_{S2}/(T_{S1}+T_{S2})$, T_{S1} – time for a testing mouse interacting with a familiar mouse (S1, Stranger 1), T_{S2} – time for a testing mouse interacting with a novel mouse (S2, Stranger 2). The duration of sniffing, defined as positioning of the nose of the test mouse within 2.5 cm of a cage, was measured using software EthoVision XT11.5 (Noldus).

3. Home cage social interaction test. The social interaction test was performed as previously described [2]. Each mouse was left alone in its home cage for 15 min. An unfamiliar male

C57BL/6N mouse of the same age was then introduced. The behavior of the test mouse was video-recorded for 10 min and scored the time of active interactions, including sniffing, allo-grooming, mounting and following.

4. Olfactory habituation/dishabituation test. This test was conducted as previously described [3]. Each subject mouse was tested in a clean mouse cage. Cotton tipped swabs were used to deliver odor stimuli. Olfactory cues were designed to measure familiar or unfamiliar odors, with or without social odors. Three identical swabs (2-min for each swab) were orderly assayed for the habituation to the same odor. Water, almond odor (prepared from almondretrieve, 1:100 dilution in tap water), banana odor (prepared from imitation banana flavor, 1:100 dilution), odor from cage 1 (social odor 1), odor from cage 2 (social odor 2) were presented in sequence to assay the dishabituation to different odors. Water, almond odor, and banana odor were prepared by dipping the cotton tip in the solution for 2 sec. Social odors were prepared by wiping a swab in pattern across a soiled cage of unfamiliar mice of the same sex. Time spent sniffing the swab was quantitated with a stopwatch by an observer. Sniffing time was scored when the distance between mouse's nose and the swab was 1 cm or shorter.

5. Self-grooming test. Mouse was placed in an empty cage without bedding. After 10-min habituation, mouse behaviors were recorded for another 10-min. Self-grooming behavior was defined as stroking or scratching of the body or face, or licking body parts. The cumulative time spent in grooming all body regions were evaluated by using a stopwatch as described previously [4].

6. Novel object recognition [5]. Short habituation session, mouse was placed into a Plexiglas rectangular cage (22 cm height × 44 cm length × 22 cm width) for 5 min. In the familiarization session (twenty-four hours after habituation session), the mouse was presented with a pair of identical objects (either towers of Lego bricks or Falcon tissue culture flasks) 5 cm away from the walls. The time when a mouse shows any investigative behaviors (head orientation or sniffing occurring, or entering an area within 1 cm around the object), is considered as exploring time. Stopwatch was used to record the time spent exploring each object until the total exploring time reached 20 seconds. During the testing trial (testing phase, performed 24 hours later), one of the familiar objects was replaced by a novel object. The

exploring time for the familiar or the novel object during the test phase was recorded until 20 seconds of total exploring time was reached.

7. Contextual fear conditioning. During training, mouse was first allowed to freely explore the apparatus (MED-VFC-NIR-M; Med Associates) for 3 min, and then exposed to 4 times of tone-foot shock pairings (tone, 30 sec, 80 dB; foot shock, 1 sec, 0.75 mA) with an interval of 80 sec. Twenty four hours after training, mouse was returned to the chamber for 2 minutes to evaluate contextual fear memory. The percentage of freezing time during training and testing was measured using Med Associates Video-Tracking and scoring software.

8. Elevated plus maze test. The test consists of an elevated, plus-sign-shaped runway that was 40 cm above the floor, with two wall-closed arms (10 × 50 cm), two open arms (10 × 50 cm) and one intersection (10 × 10 cm). Mouse was allowed to acclimate to the testing room 1 hour before the test. Then, each mouse was placed in the center of the EPM, facing the closed arm, and was videotaped for 5 min. The time spent in the closed or open arms was quantified using software EthoVision XT 11.5 (Noldus).

9. Open field test. The open field test was performed in a rectangular chamber (60 × 60 × 40 cm) that was made of gray polyvinyl chloride and was monitored by an automated video tracking system. The center area was illuminated by 25 W halogen bulbs (200 cm above field). The mouse was gently placed in the center for 30 min. After each trial, the apparatus was swept out with water that contained 0.1% acetic acid. The the distance moved were automatically calculated using the DigBehv animal behavior analysis program (MED-VFC-NIR-M; Med Associates).

10. Seizure susceptibility test. Pentylenetetrazol (PTZ) (SIGMA, USA) was dissolved in physiological saline and administered intraperitoneally to the wild type mice and *Htr3a*$^{-/-}$ mice (8-9 weeks old males; 20 g-27 g body weight) at a dose of 60 mg per kg body weight in a total volume of 0.20-0.25 ml. The mice were monitored and video-recorded in a clean cage for 1 hour. The behavioral indicators of seizure activity were as follows [6]: (1) hypoactivity (abdomen in full contact with the bottom of the cage in the resting position); (2) focal clonus (of face, head, or forelimbs); (3) clonus (rearing, falling, and clonus of four limbs and tail); (4) clonic (tonic seizure, tonic hindlimb extension, or death).

11. Memantine treatment. Memantine was purchased from SIGMA (USA) and dissolved in saline. Memantine treatment was conducted as previously described [7]. Memantine (5 mg/kg) or saline alone (control) was administered to mice by intraperitoneal (i.p.) injection 30 min before behavior tests, such as the social interaction test, social approach task, measurements of repetitive self-grooming behaviors or the injection of PTZ. For behavior tests that require training session and test session, such as novel object recognition test and contextual fear conditioning test, memantine (5 mg/kg) or saline was i.p. injected into mice 30 min before the training session and 30 min before test session. Behavior tests were performed as described above. Electrophysiological recordings were performed as described above, with/without the bath containing 1 μM memantine. The current changes were evaluated before, and 5 min after application of memantine.

RNA-seq and differential expression analysis

Each RNA sample was extracted from dissected hippocampi of adult mice according to the manufacturer's protocol (RNAeasy Mini Kit, Qiagen, USA). The quality and yield of the isolated RNAs were assessed using a NanoDrop Spectrophotometer (Thermo Fisher Scientific, Waltham, MA, USA) and Agilent 2100 Bioanalyzer (Agilent Technologies, Santa Clara, CA, USA). Only RNAs with a high RNA integrity number (RIN > 9) were selected and used for the subsequent sequencing. RNA sequencing was performed at Novogene (Beijing, China) using Illumina NovaSeq. The paired-end reads were aligned to the reference mice genome (mm10 assembly) and low-quality regions were removed. A Picard tool, MarkDuplicates, was used to mark duplicate reads. Reference genome (mm10) and annotation files were downloaded from UCSC Genome Browser. Reads numbers mapped to each gene were counted using HTseq-count (v0.9.0) [8]. Genes with counts > 4 counts in at least 4 of 6 samples were defined as expressed genes in the analysis with DESeq2 (v1.20.0) [9]. Genes with CPM (count-per-million) >1 in at least one of six samples were considered as expressed genes in the analysis with edgeR (v3.22.5) [10]. Hippocampal expressed genes are intersection of the expressed genes identified by DESeq2 and edgeR. Heatmap of differentially expressed genes (DEGs) and principal component analysis (PCA) were carried out based on regularized log2-transformed data using pheatmap package and plotPCA in DESeq2. Differential expression analysis on two groups was performed using the DESeq2 and the edgeR (v3.22.5) [10], using a cutoff of FDR < 0.05 for DESeq2

and $p < 0.01$ for edgeR. Differentially expressed genes (DEGs) was the intersection part of DESeq2 ($adj.p < 0.05$) and edgeR ($p < 0.01$).

Functional enrichment analyses for differentially expressed genes

1. Functional annotations of up-regulated and down-regulated genes were done using Database for Annotation, Visualization and Integrated Discovery (DAVID) [11] tools (v6.8) and terms were identified with FDR less than 0.05. Visualization and plot of top selected terms were done using ggplot2 package (v3.0.0).
2. Data source for genes used for enrichment analyses: Autism candidate genes from AutDB [12]; epilepsy candidate genes from HGMD [13], OMIM [14], PhenGenI [15], and keyword is 'epilepsy' for three databases; learning/memory related genes from KEGG and PubMed. Human gene names were converted to orthologous mouse genes using the Ensemble BioMart [16]. The *p*-values of enrichment of disease-related genes in DEGs was calculated using Fisher's Exact Test. The same method was used for up-regulated genes and down-regulated genes.

Construction of hippocampal interactome and DEG Network

We constructed a hippocampal interactome by mapping 16,435 expressed genes from the mouse hippocampal transcriptome to the whole mouse interactome from BioGRID [17], which contains 4,353 nodes and 9,618 edges. We mapped the 2,092 DEGs to the mouse hippocampal interactome to retrieve hippocampal DEG Network containing DEGs and their first co-expressed neighbors. Co-expression relationship was determined by a cutoff 0.75 of correlation coefficient, which was calculated based on FPKM value (fragments per kilobase of transcript per million mapped reads) using WGCNA [18]. Self-loop edges and zero-degree nodes were removed.

Networks for autism, epilepsy and learning/memory

We mapped the 1,036 ASD candidate genes to the mouse hippocampal interactome to retrieve an ASD Network containing ASD candidate genes and their first co-expressed neighbors. The same method was also applied for retrieving epilepsy Network and learning/memory Network.

References

1. Moy SS, Nadler JJ, Perez A, Barbaro RP, Johns JM, Magnuson TR, et al. Sociability and preference for social novelty in five inbred strains: an approach to assess autistic-like behavior in mice. Genes Brain Behav. 2010; 3: 287-302.
2. Sato A, Kasai S, Kobayashi T, Takamatsu Y, Hino O, Ikeda K, et al. Rapamycin reverses impaired social interaction in mouse models of tuberous sclerosis complex. Nat Commun. 2012; 3: 1292.
3. Yang M, Crawley JN. Simple behavioral assessment of mouse olfaction. Curr Protoc Neurosci. 2009; Chapter 8: Unit 8.24.
4. Mcfarlane HG, Kusek GK, Yang M, Phoenix JL, Bolivar VJ, Crawley JN. Autism-like behavioral phenotypes in BTBR T+tf/J mice. Genes Brain Behav. 2010; 7: 152-63.
5. Marianne L, Anne Q, Valentine B, Benoît H, Michel B, Pascale SB, et al. Object recognition test in mice. Nat Protoc. 2013; 8: 2531-7.
6. Zhou L, Zhou L, Su LD, Cao SL, Xie YJ, Wang N, et al. Celecoxib Ameliorates Seizure Susceptibility in Autosomal Dominant Lateral Temporal Epilepsy. J Neurosci. 2018; 38: 3346-57.
7. Kang J, Eunjoon K. Suppression of NMDA receptor function in mice prenatally exposed to valproic acid improves social deficits and repetitive behaviors. Front Mol Neurosci. 2015; 8: 17.
8. Anders S, Pyl PT, Huber W. HTSeq--a Python framework to work with high-throughput sequencing data. Bioinformatics. 2015; 31: 166-9.
9. Love MI, Huber W, Anders S. Moderated estimation of fold change and dispersion for RNA-seq data with DESeq2. Genome Biol. 2014; 15: 550.
10. Robinson MD, McCarthy DJ, Smyth GK. edgeR: a Bioconductor package for differential expression analysis of digital gene expression data. Bioinformatics. 2010; 26: 139-40.
11. Huang da W, Sherman BT, Lempicki RA. Systematic and integrative analysis of large gene lists using DAVID bioinformatics resources. Nat Protoc. 2009; 4: 44-57.
12. Pereanu W, Larsen EC, Das I, Estevez MA, Sarkar AA, Spring-Pearson S, et al. AutDB: a platform to decode the genetic architecture of autism. Nucleic Acids Res. 2018; 46: D1049-D54.
13. Stenson PD, Ball EV, Mort M, Phillips AD, Shiel JA, Thomas NS, et al. Human Gene Mutation Database (HGMD): 2003 update. Hum Mutat. 2003; 21: 577-81.
14. Amberger JS, Bocchini CA, Schiettecatte F, Scott AF, Hamosh A. OMIM.org: Online Mendelian Inheritance in Man (OMIM®), an online catalog of human genes and genetic disorders. Nucleic Acids Res. 2015; 43: D789-98.
15. Ramos EM, Hoffman D, Junkins HA, Maglott D, Phan L, Sherry ST, et al. Phenotype-Genotype Integrator (PheGenI): synthesizing genome-wide association study (GWAS) data with existing genomic resources. Eur J Hum Genet. 2014; 22: 144-7.
16. Smedley D, Haider S, Ballester B, Holland R, London D, Thorisson G, et al. BioMart--biological queries made easy. BMC Genomics. 2009; 10: 22.
17. Oughtred R, Stark C, Breitkreutz BJ, Rust J, Boucher L, Chang C, et al. The BioGRID interaction database: 2019 update. Nucleic Acids Res. 2019; 47: D1.
18. Langfelder P, Horvath S. WGCNA: an R package for weighted correlation network analysis. BMC Bioinformatics. 2008; 9: 559.

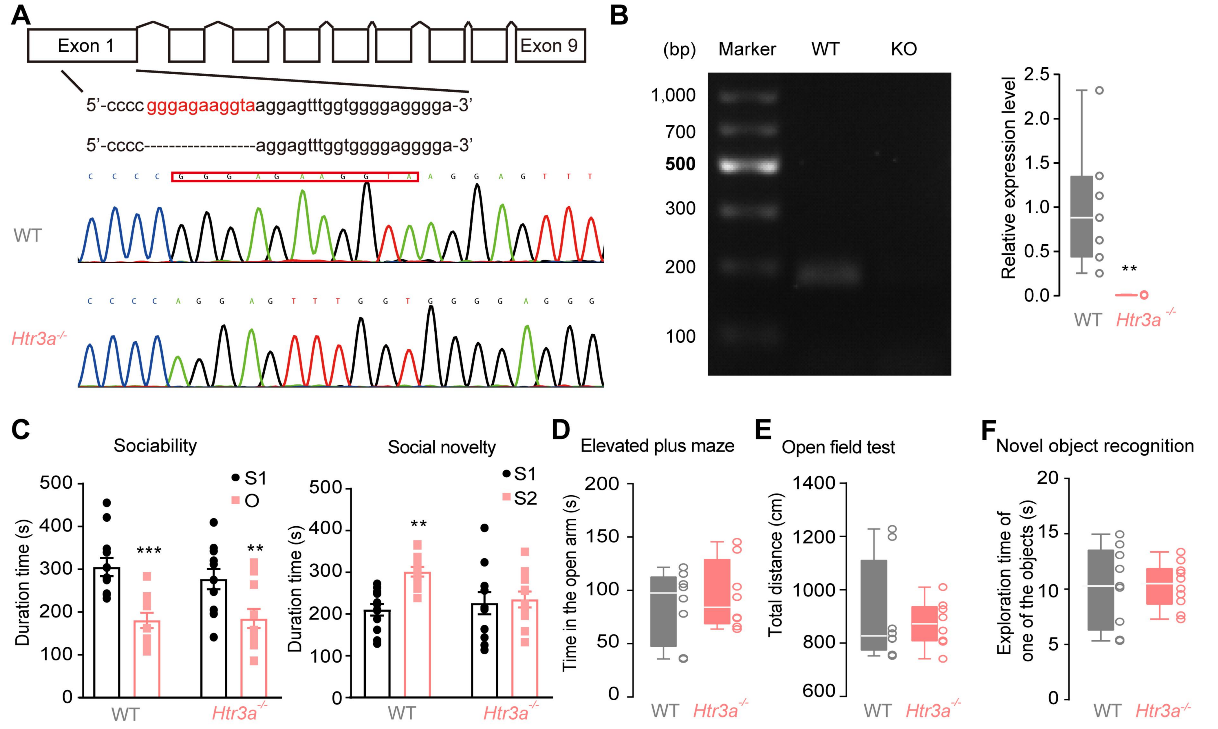

Figure S1. Genotyping and behavioral deficits.

(A) Mutant offspring were identified by PCR and Sanger sequencing.

(B) Ablation of mutant sites in *Htr3a* in the hippocampus of *Htr3a*$^{-/-}$ mice were identified by RT-PCR and qRT-PCR. The sizes (bp) of the DNA ladder are labeled on the left.

(C) **Sociability (left bar plot):** In the 10-min sociability phase of the social approach task, both *Htr3a*$^{-/-}$ and WT mice showed preference to interact with a stranger mouse (S1, Stranger1) rather than an empty cage (O, Object) (two-way ANOVA test, p = 0.0002, n = 12 for WT mice, p = 0.0090, n = 11 for *Htr3a*$^{-/-}$ mice). **Social novelty (right bar plot):** In the 10-min social novelty phase of the social approach task, *Htr3a*$^{-/-}$ mice showed no significant preference to interact with a stranger mouse (S2, Stranger 2) over a familiar mouse (S1, Stranger 1), while WT mice showed preference to interact with the stranger mouse (S2) over the familiar mouse (S1) (two-way ANOVA test, p = 0.0017, n = 12 for WT mice; p = 0.9308, n = 11 for *Htr3a*$^{-/-}$ mice).

(D) In the elevated plus maze test, *Htr3a*$^{-/-}$ and WT mice showed no difference of duration time in open arms (Student's t test, p = 0.8526, n = 8 for WT mice, n = 12 for *Htr3a*$^{-/-}$ mice).

(E) *Htr3a*$^{-/-}$ mice showed normal locomotor activity in the 5-min open field test (Student's t test, p = 0.2714, n = 8 mice for each group).

(F) In the training phase of the novel object recognition test, *Htr3a*$^{-/-}$ and WT mice showed no preference for the object (Student's t test, p = 0.9353, n = 9 for WT mice, n = 10 for *Htr3a*$^{-/-}$ mice).

Data are presented as boxplots (median and 5th-95th percentile whiskers), or as mean ± SEM., * $p < 0.05$; ** $p < 0.01$, *** $p < 0.001$.

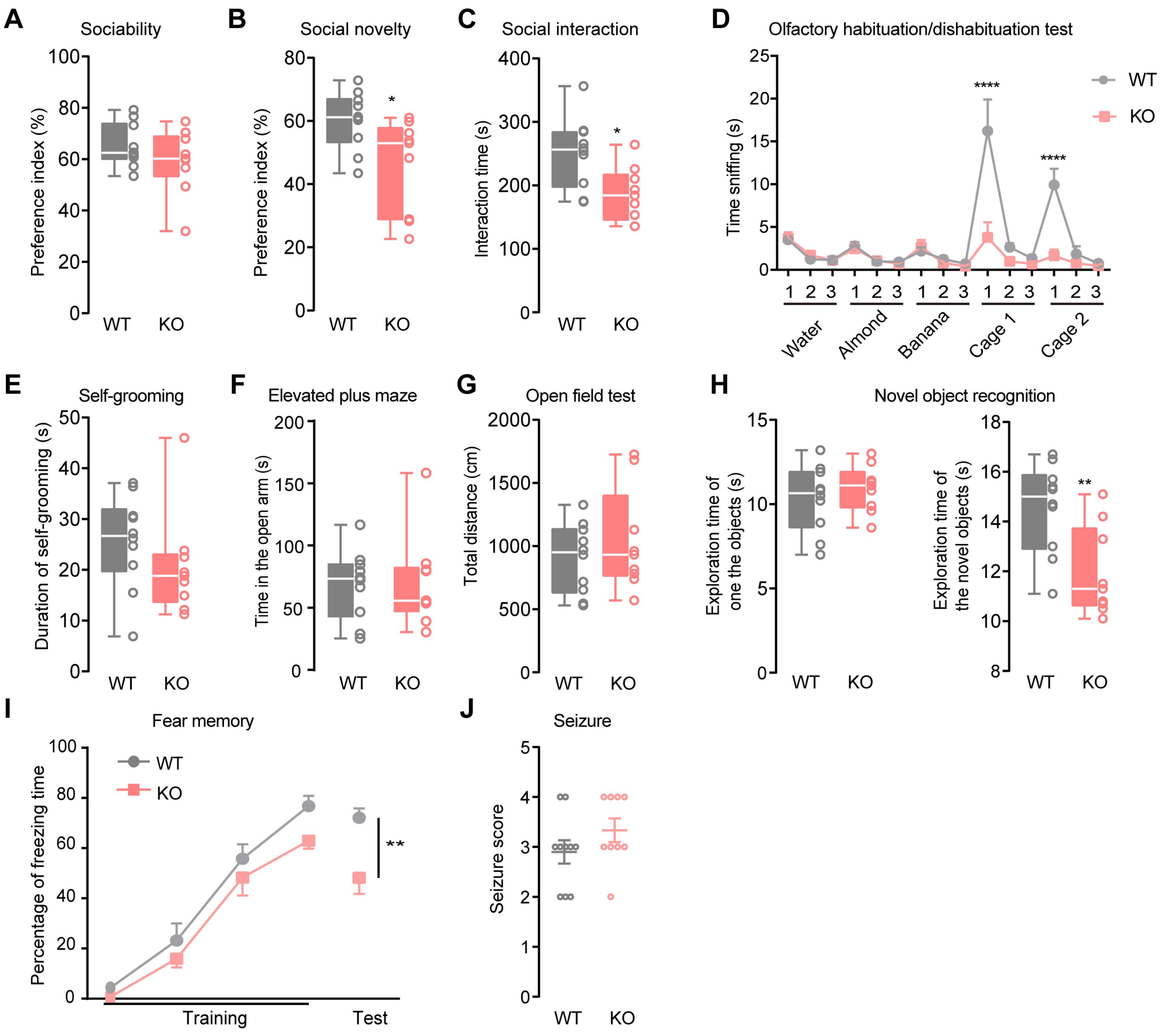

Figure S2. Female *Htr3a* KO mice exhibited impaired social behavior and memory.

(A) In the 10-min sociability phase of the social approach task, there was no significant difference between female knockout and WT mice (S1, Stranger1; O, Object) (Student's *t* test, $p = 0.2426$).

(B) Compared with WT mice, female *Htr3a*$^{-/-}$ mice showed significantly decreased preference to interact with a stranger mouse (S2, Stranger 2) over a familiar mouse (S1, Stranger 1) in the 10-min social novelty phase of the social approach task (Student's *t* test, $p = 0.0198$).

(C) Compared with WT mice, female *Htr3a*$^{-/-}$ mice spent less time interacting with a stranger mouse in the home cage social interaction test (Student's *t* test, $p = 0.0116$).

(D) Social and non-social odors were presented to female mice three times in the olfactory habituation/dishabituation test. Female *Htr3a*$^{-/-}$ mice spent less time sniffing social odors compared to WT mice. Statistic tests: (1) two-way ANOVA, odor effect $F_{14,255} = 14.09$, $p < 0.0001$; genotype effect $F_{1,255} = 21.94$, $p < 0.0001$; interaction between odor and genotype $F_{14,255} = 7.421$, $p < 0.0001$; (2) Bonferroni's multiple comparisons test, $p < 0.0001$ for the first test of the mouse cage 1; $p < 0.0001$ for the first test of mouse cage 2.

(E) Female *Htr3a*$^{-/-}$ and WT mice spent a similar amount of time on self-grooming (Mann Whitney test, $p = 0.1128$).

(F) In the EPM test, female *Htr3a*$^{-/-}$ and WT mice showed no difference of duration time in open arms (Student's *t* test, $p = 0.8742$).

(G) Female *Htr3a*$^{-/-}$ mice showed normal locomotor activity in 5-min open field test (Student's *t* test, $p = 0.4041$).

(H) In the training phase of the novel object recognition test, *Htr3a*$^{-/-}$ and WT mice showed no preference for the object (Student's *t* test, $p = 0.4579$). In the novel object recognition test phase, *Htr3a*$^{-/-}$ mice spent less time exploring the novel object than WT mice (Student's *t* test, $p = 0.0060$).

(I) In the contextual fear conditioning test, KO mice showed a lower percentage of freezing time in the 4 times of the tone-shock paired training (two-way ANOVA test, $F_{1,76} = 5.551$, $p = 0.0210$). After 24 hours, female knockout mice showed less freezing time in the contextual fear memory test than WT mice (Student's *t* test, $p = 0.0035$).

(J) Female *Htr3a*$^{-/-}$ mice showed a normal seizure state after PTZ injection (Mann Whitney test, $p = 0.2445$).

$n = 10$ for WT mice, $n = 9$ for *Htr3a*$^{-/-}$ mice; data are presented as boxplots (median and 5th-95th percentile whiskers), or as mean ± SEM., * $p < 0.05$; ** $p < 0.01$

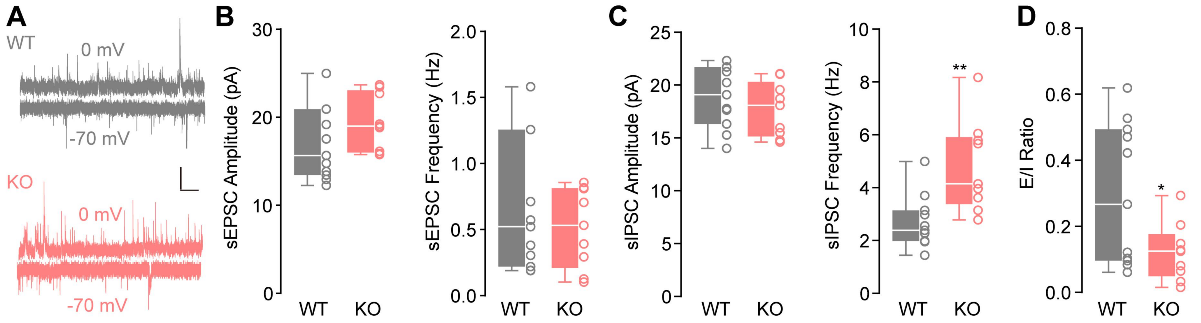

Figure S3. Enhanced GABAergic transmission perturbed excitatory / inhibitory balance.

(A) Representative traces of sEPSCs (held at -70 mV) and sIPSCs (held at 0 mV) recorded on the same neuron in the *Htr3a*$^{-/-}$ and WT mice. Scale bar: 20 pA, 2 s.

(B) Boxplots of sEPSC amplitude and frequency, showing normal sEPSC amplitude (Student's *t* test, $p = 0.2673$) and frequency (Student's *t* test, $p = 0.4711$) in *Htr3a*$^{-/-}$ and WT mice.

(C) Boxplots of sIPSC amplitude and frequency, showing normal sIPSC amplitude (Student's *t* test, $p = 0.3627$) and increased sIPSC frequency (Student's *t* test, $p = 0.0029$) in *Htr3a*$^{-/-}$ and WT mice.

(D) Boxplots of the ratio of sEPSC / sIPSC frequency, showing decreased E/I ratio in *Htr3a*$^{-/-}$ mice (Student's *t* test, $p = 0.0328$)

$n = 11$ cells from 3 WT mice, $n = 9$ cells from 3 *Htr3a*$^{-/-}$ mice; median and 5th-95th percentile whiskers, or as mean ± SEM., * $p < 0.05$; ** $p < 0.01$, *** $p < 0.001$.

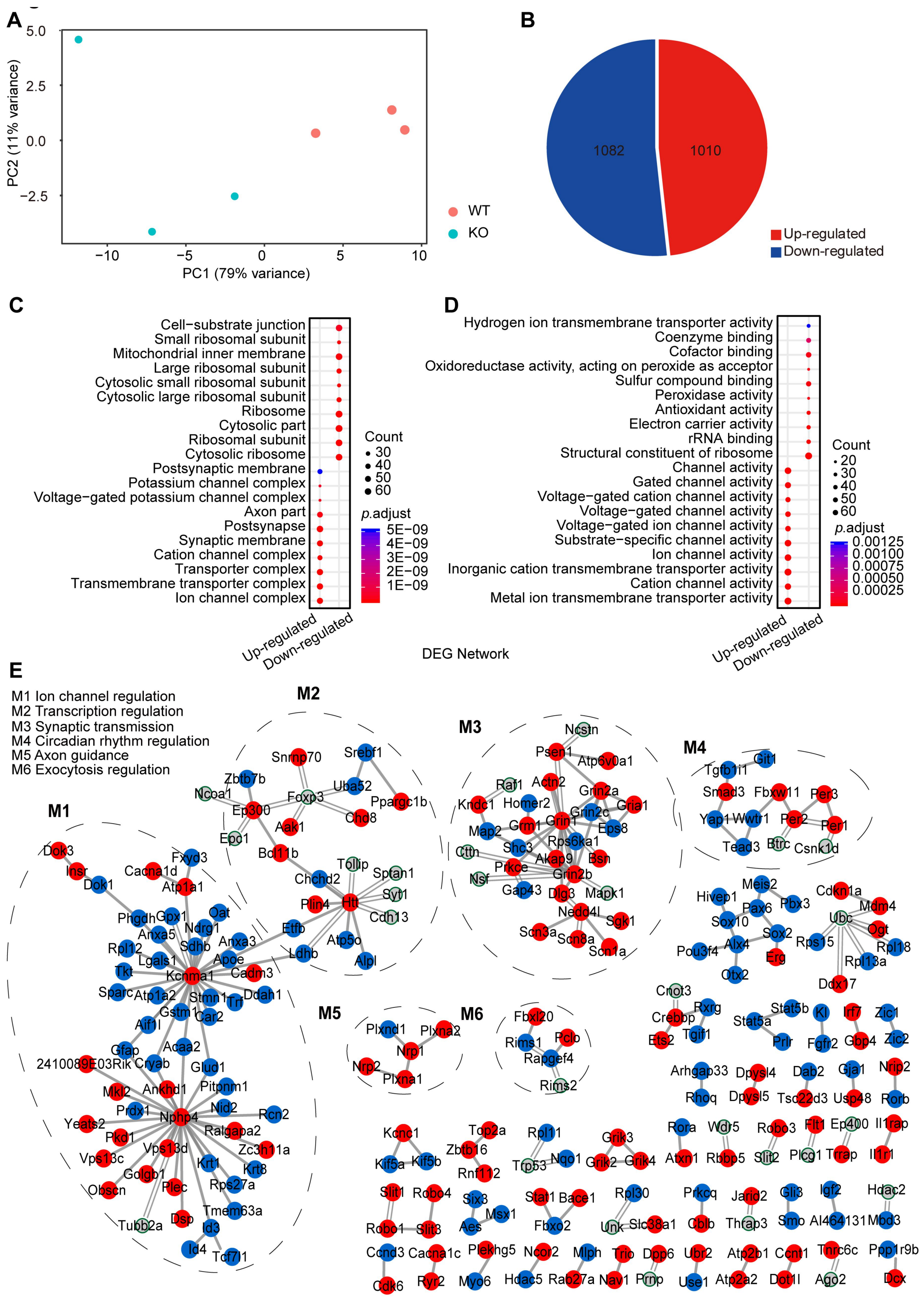

Figure S4. Transcriptome sequencing and DEG Network.

(A) PCA was performed on the regularized log2-transformed data using plotPCA DEseq2.

(B) The Pie chart shows 1,010 up-regulated genes and 1,082 down-regulated genes in *Htr3a* KO mice.

(C-D) Top 10 cellular components **(C)**, molecular functions **(D)** in Gene Ontology (GO) of the up-regulated and down-regulated genes are listed as derived from the DAVID functional annotation tool with an adjusted *p* value cutoff of 0.05 (Table S3).

(E) The protein interaction network for DEGs (DEG Network) consists of 245 nodes and 222 edges. Dotted circles indicate the subnetworks (the major components of the network). Each of these subnetworks is enriched with indicated function, and is thus considered to be a functional module (marked as M1-6). Red node: upregulated; blue node: downregulated; gray node: without expression change; node with green border: co-expressed neighbor; gray line: protein-protein interaction (PPI); double lines: PPI and co-expression.

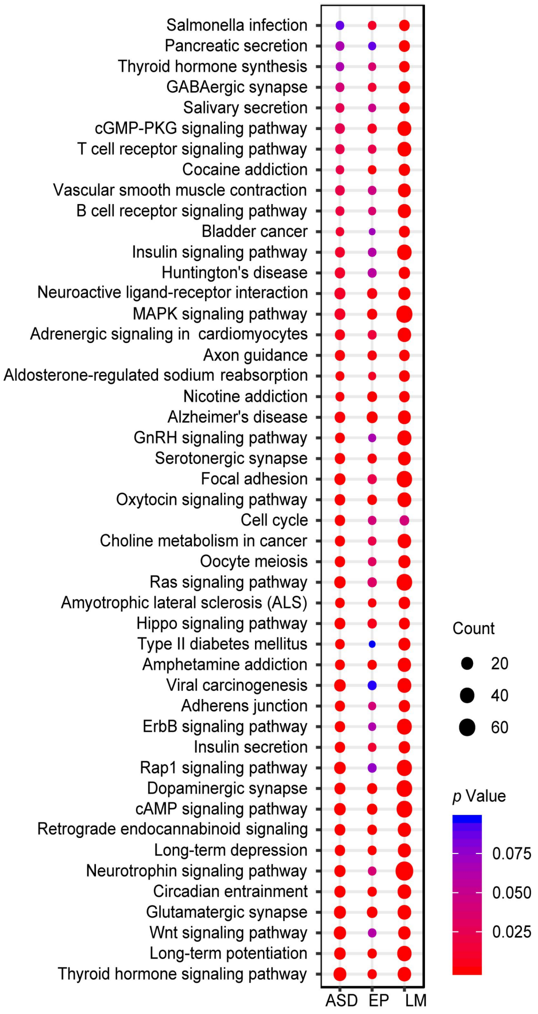

Figure S5. Comparison of the enriched pathways between ASD-, EP- and LM Networks.

There were 47 enriched pathways shared by three networks. The enrichment analysis was performed using DAVID functional annotation tool with an adjusted *p*-value cutoff 0.05.

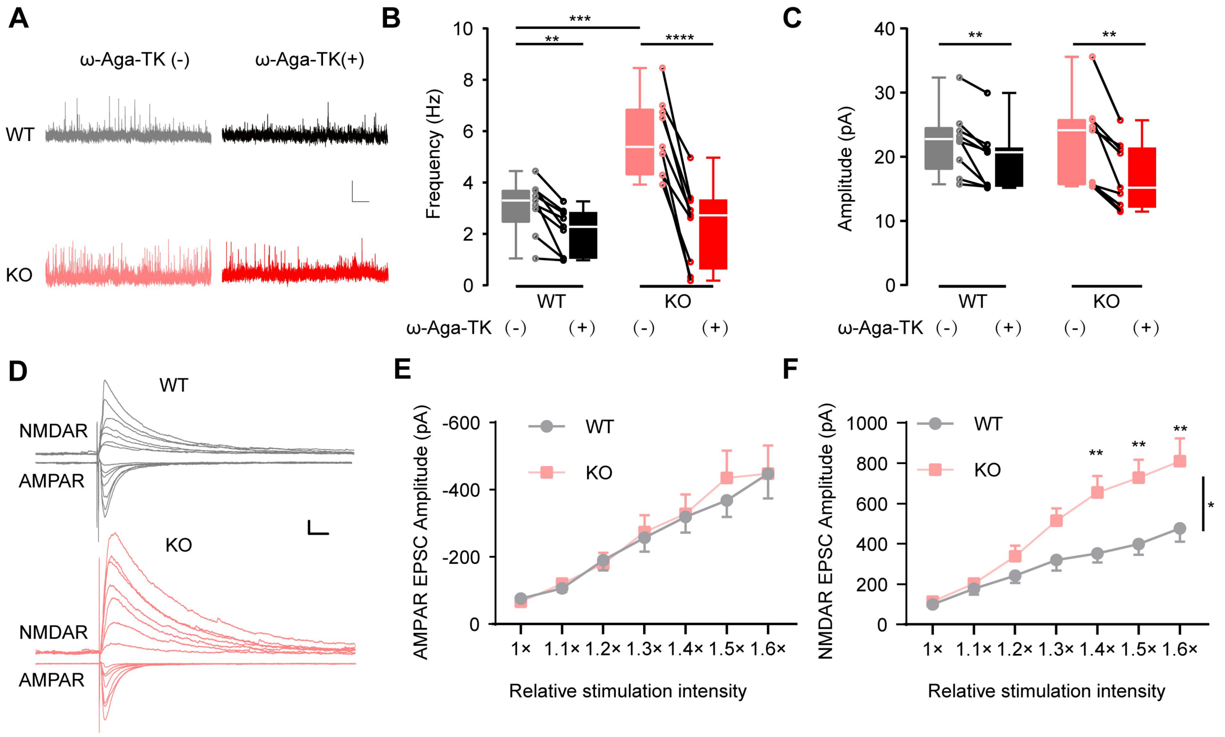

Figure S6. The P/Q calcium channel mediated the GABAergic transmission and the NMDAR current were enhanced in the pyramidal neurons in the CA1 region of *Htr3a*$^{-/-}$ mice.

(A-C) The antagonist of P/Q calcium channel, ω-Aga-TK reduced sIPSC. Representative traces of sIPSCs recorded in *Htr3a*$^{-/-}$ and WT mice, scale bar, 20 pA, 2 s (**A**). Treatment with ω-Aga-TK significantly decreased sIPSC frequency (**B**) and amplitude (**C**) in *Htr3a*$^{-/-}$ mice (For frequency, $WT_{(-)}$ vs. $KO_{(-)}$, Student's *t* test $p = 0.0005$, $WT_{(-)}$ vs. $WT_{(+)}$, Paired *t* test $p = 0.0018$, $KO_{(-)}$ vs. $KO_{(+)}$, Paired *t* test $p < 0.0001$, $WT_{(-)}$ vs. $KO_{(+)}$, Student's *t* test $p = 0.2804$; for amplitude, $WT_{(-)}$ vs. $KO_{(-)}$, Student's *t* test $p = 0.9116$, $WT_{(-)}$ vs. $WT_{(+)}$, Paired *t* test $p = 0.0041$, $KO_{(-)}$ vs. KO-(+), Paired *t* test $p = 0.0018$; $n = 9$ cells from 3 mice for each group).

(D) Representative traces of the evoked AMPAR current (held at -70 mV) and NMDAR current (held at +40 mV) of pyramidal neurons in the CA1 region of the *Htr3a*$^{-/-}$ and WT mice. Scale bar: 20 pA, 100 ms.

(E) Normal AMPAR current in pyramidal neurons in the CA1 region of *Htr3a*$^{-/-}$ mice. (For AMPAR current, two-way ANOVA, $F_{1, 140} = 0.1517$, $p = 0.6975$; $n = 10$ cells from 3 WT mice, $n = 12$ cells from 4 KO mice).

(F) Increased NMDAR current in pyramidal neurons in the CA1 region of *Htr3a*$^{-/-}$ mice. (two-way ANOVA, $F_{1, 140} = 28.61$, $p < 0.0001$. $n = 10$ cells from 3 WT mice, $n = 12$ cells from 4 KO mice).

Data are presented as boxplots (median and 5th-95th percentile whiskers), or as mean ± SEM., * $p < 0.05$; ** $p < 0.01$, *** $p < 0.001$, **** $p < 0.0001$.
